# Chromatin Modifier EP400 Regulates Oocyte Quality and Zygotic Genome Activation in Mice

**DOI:** 10.1002/advs.202308018

**Published:** 2024-03-17

**Authors:** Qing Tian, Ying Yin, Yu Tian, Yufan Wang, Yong‐feng Wang, Rikiro Fukunaga, Toshihiro Fujii, Ai‐hua Liao, Lei Li, Wei Zhang, Ximiao He, Wenpei Xiang, Li‐quan Zhou

**Affiliations:** ^1^ Institute of Reproductive Health Tongji Medical College Huazhong University of Science and Technology Wuhan Hubei 430030 China; ^2^ Department of Gynecology and Obstetrics Zhongnan Hospital of Wuhan University Wuhan Hubei 430071 China; ^3^ Department of Physiology School of Basic Medicine, Tongji Medical College Huazhong University of Science and Technology Wuhan Hubei 430030 China; ^4^ Center for Genomics and Proteomics Research School of Basic Medicine, Tongji Medical College Huazhong University of Science and Technology Wuhan Hubei 430030 China; ^5^ Hubei Key Laboratory of Drug Target Research and Pharmacodynamic Evaluation Huazhong University of Science and Technology Wuhan Hubei 430030 China; ^6^ Department of Biochemistry Osaka Medical and Pharmaceutical University Takatsuki Osaka 569‐1094 Japan; ^7^ State Key Laboratory of Stem Cell and Reproductive Biology Institute of Zoology Chinese Academy of Sciences Beijing 100101 China

**Keywords:** EP400, H3.3, oocyte, preimplantation, zygotic genome activation

## Abstract

Epigenetic modifiers that accumulate in oocytes, play a crucial role in steering the developmental program of cleavage embryos and initiating life. However, the identification of key maternal epigenetic regulators remains elusive. In the findings, the essential role of maternal *Ep400*, a chaperone for H3.3, in oocyte quality and early embryo development in mice is highlighted. Depletion of *Ep400* in oocytes resulted in a decline in oocyte quality and abnormalities in fertilization. Preimplantation embryos lacking maternal *Ep400* exhibited reduced major zygotic genome activation (ZGA) and experienced developmental arrest at the 2‐to‐4‐cell stage. The study shows that EP400 forms protein complex with NFYA, occupies promoters of major ZGA genes, modulates H3.3 distribution between euchromatin and heterochromatin, promotes transcription elongation, activates the expression of genes regulating mitochondrial functions, and facilitates the expression of rate‐limiting enzymes of the TCA cycle. This intricate process driven by *Ep400* ensures the proper execution of the developmental program, emphasizing its critical role in maternal‐to‐embryonic transition.

## Graphical abstract

Maternal *Ep400*, a chaperone for H3.3, is required for oocyte quality and early embryo development in mice. *Ep400* depletion in oocytes causes oocyte quality decline and fertilization abnormity. In preimplantation embryos, EP400 occupies promoters of major ZGA genes, modulates H3.3 distribution, and promotes transcription elongation. Preimplantation embryos without maternal *Ep400* have reduced transcription activity and developmental arrest at 2‐to‐4‐cell stage.

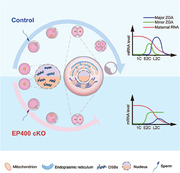

## 1 Introduction

Throughout oogenesis, oocytes undergo continuous expansion, accumulating a substantial reservoir of mRNAs and proteins. This accumulation is vital to support the maternal‐to‐embryonic transition, encompassing critical processes such as meiosis, fertilization, zygotic genome activation (ZGA), and early embryonic development. The developmental potency and health of offspring significantly depend on the quality of both oocytes and cleavage‐stage embryos. The quality control is orchestrated by maternally supplied chromatin reprogramming factors, overseeing genome‐wide epigenetic modifications. Despite the paramount importance of these regulators, their identification has proven challenging due to a scarcity of materials and the intricacies of genetic manipulation.

Histone variants, evolving gradually with distinct functions from canonical histones, contribute to this intricate process.^[^ ^1^ ^]^ H3.3, a variant of histone H3 distinguished by just five amino acids from canonical histone H3.1/H3.2, integrates into the genome in a replication‐independent manner.^[^ ^2^ ^]^ During oogenesis, the pronounced accumulation of H3.3 around transcriptional start sites (TSSs) throughout the genome diminishes, ultimately leading to an even distribution of H3.3 across the genome in MII oocytes. This unconventional distribution persists until the 2‐cell stage and then rapidly transits to a canonical distribution pattern in a DNA replication‐dependent manner.^[^ ^3^ ^]^ In growing oocytes, the continual replacement of H3.3 serves to safeguard the genome against heightened chromatin accessibility and DNA damage, facilitating the establishment of *de novo* DNA methylation.^[^ ^4^ ^]^ Upon fertilization, the histone chaperone HIRA plays a crucial role in facilitating incorporation of maternal‐stored H3.3 onto the male genome after removal of protamines. This process is essential for stimulating rRNA transcription and DNA replication in zygotes.^[^ ^5^ ^]^ Additionally, ASF1 contributes to this chromatin assembly by loading the H3.3‐H4 dimer onto the HIRA complex, enhancing the efficiency of HIRA‐mediated nucleosome assembly.^[^ ^6^ ^]^ The involvement of other histone chaperones in chromatin assembly upon fertilization remains unknown. Moreover, there is growing interests in understanding the roles of H3.3 in chromatin condensation status and the formation of pericentromeric heterochromatin, which is medicated by DAXX in early embryos.^[^ ^7^ ^]^ Notably, the knockdown of H3.3 in mouse zygotes leads to developmental arrest before the blastocyst stage. However, the introduction of exogenous H3.3, as opposed to canonical H3.1, successfully rescues the developmental failure. This underscores the essential and non‐redundant roles that H3.3 plays in the developmental competence of early embryos.^[^ ^7a^ ^]^ Despite this understanding, the specific contributions of H3.3 and its chaperones to the transcriptional program and developmental control in cleavage‐stage embryos remain elusive.

EP400 was initially identified as an E1A binding protein characterized by an SWI2/SNF2‐type ATPase/helicase domain, a SANT domain, and a glutamine‐rich (Q‐rich) domain.^[^ ^8^ ^]^ Afterwards, EP400 was found to function as an H3.3 chaperone, promoting gene expression and mediating the integration of H3.3 into the promoters and enhancers of active genes.^[^ ^9^ ^]^ Notably, EP400 plays a crucial role in promoting double‐strand break (DSB) repair through homologous recombination. It forms protein complex with TIP60, catalyzing histone acetylation around DSBs. This modification leads to the establishment of an open chromatin state, facilitating the recruitment of DSB repair proteins such as Ku70 and Ku80.^[^ ^10^ ^]^ Furthermore, the TIP60‐EP400 complex is involved in catalyzing γH2A.X acetylation at DSB sites, which in turn promotes the replacement of γH2A.X with newly synthesized H2A.X.^[^ ^11^ ^]^ Additionally, EP400 directly interacts with key DSB repair proteins including ATM, BRCA1, and RAD51.^[^ ^12^ ^]^ For instance, EP400 recruits BRCA1 and 53BP1 to DSB sites, thereby promoting RNF8‐mediated chromatin ubiquitination.^[^ ^12b^ ^]^ Beyond its role in DSB repair, EP400 also contributes to the clearance of reactive oxide species (ROS) and prevents cell senescence through the P53‐P21 pathway.^[^ ^13^ ^]^ Knockdown of *Ep400* in cells results in DSB repair defect, cell cycle arrest, and an increase in apoptosis.^[^ ^12^, ^13^, ^14^ ^]^

Recent studies have unveiled an expanded role for *Ep400* in transcriptional regulation.^[^ ^8b^ ^]^ In mouse embryonic stem cells (ESCs), EP400 occupies the promoters of ≈55% of genes throughout the entire genome. Its distribution pattern demonstrates positive correlation with RNA polymerase II (Pol II) and H3K4me3, while displaying a negative correlation with H3K27me3.^[^ ^15^ ^]^ The recruitment of EP400 onto chromatin is facilitated by histone deacetylase HDAC6.^[^ ^16^ ^]^ Furthermore, it has been observed that active transcription activity enhances the chromatin binding of the TIP60‐EP400 complex, thereby inhibiting the binding of the polycomb repressive complex 2 (PRC2).^[^ ^17^ ^]^ The TIP60‐EP400 complex is not only involved in transcription regulation, but also plays a crucial role in maintaining ESC identity by localizing to the promoters of both silent and active genes.^[^ ^15b^ ^]^ Deficiency of *Ep400* in ESCs has been reported to increase the ratio of 2‐cell‐like ESCs.^[^ ^18^ ^]^ Despite of these findings, the orchestration of H3.3 activity by EP400 in early embryos and the regulatory mechanisms by which EP400 governs maternal‐to‐embryonic transition remain unknown.

In our study, we investigated the role of *Ep400* in mouse oocyte and early embryo development through the use of a conditional knockout (cKO) mouse model. Our findings revealed that the depletion of maternal *Ep400* from mouse growing oocytes led to a decrease in oocyte quality and failure of preimplantation embryo development.

## 2 Results

### 2.1 Oocyte‐Specific Knockout of *Ep400* Dampened Female Fertility

**Figure 1:**
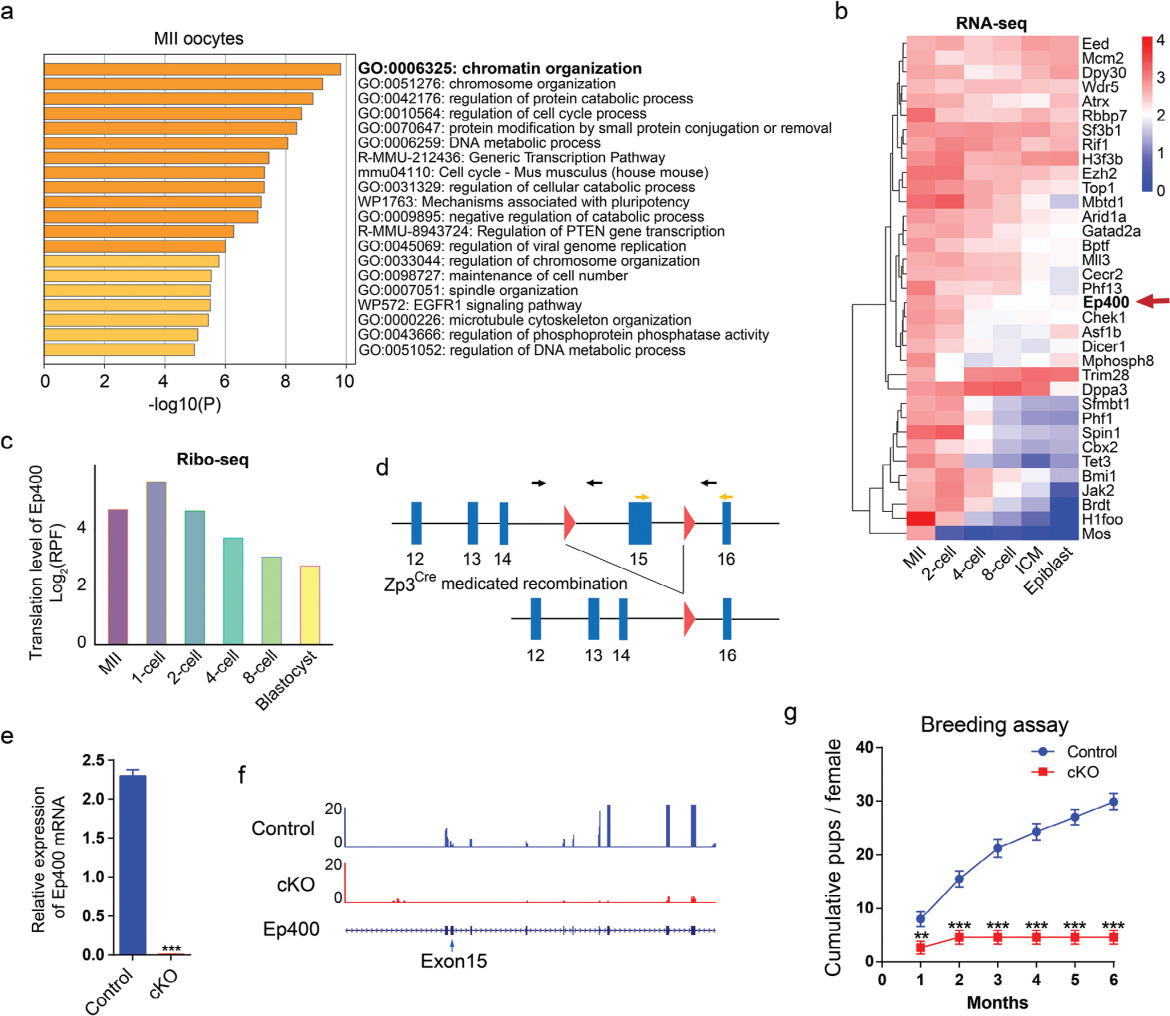
Fertility decline of *Ep400* conditional knockout female mice. a) Gene ontology analysis of the top 500 genes enriched in mouse MII oocytes. Public RNA‐seq dataset GSE22182 was used for analysis. b) Expression (log_2_FPKM) dynamics of the 35 genes involved in gene ontology term “chromatin organization” (a) in mouse MII oocytes and preimplantation embryos, with *Ep400* gene indicated by red arrow. c) Ribo‐seq result of *Ep400* gene indicates translation activities of *Ep400* mRNA during early mouse embryogenesis. Public Ribo‐seq dataset GSE165782 was used for analysis. d) Schematic description of *Ep400* depletion strategy. Black and yellow arrows indicate the positions of primer pairs used for genotyping and qRT‐PCR respectively. Red triangles represent loxP cassettes. e) Relative level of *Ep400* mRNA in GV oocytes from control and cKO mice by qRT‐PCR. Data are presented as means ± SD (*n* = 3). Two‐tailed student's *t*‐test was used to calculate *p* values. ∗∗∗ *p* < 0.001. f) Genome browser screen shot of low‐input RNA‐seq signals of mouse GV oocytes at *Ep400* locus by IGV. The result indicates that deletion of exon 15 led to instability of the whole *Ep400* transcript. g) Continuous breeding assay using 3‐month‐old female mice showed the cumulative number of pups per female for 6 months. 6 mice of each genotype were used. Data are presented as means ± SD. Two‐tailed student's *t*‐test was used to calculate *p* values. ∗∗ *p* < 0.01; ∗∗∗ *p* < 0.001.

To identify potential maternal epigenetic regulators influencing the quality of oocytes and early embryo development during maternal‐to‐embryonic transition,^[^ ^19^ ^]^ we utilized a mouse MII oocyte RNA‐seq dataset. The top 500 genes, ranked by normalized expression levels, were subjected to gene ontology analysis,^[^ ^20^ ^]^ revealing “chromatin organization” as the predominant enriched gene ontology term (**Figure** **1**a). We then examined the expression dynamics of the 35 genes associated with this gene ontology term in oocytes and early embryos, highlighting several genes with known pivotal roles in early embryonic development, including *Chek1*,^[^ ^21^ ^]^ *Dppa3*,^[^ ^22^ ^]^ *Tet3* ^[^ ^23^ ^]^ (Figure 1b). Notably, we observed that the chromatin regulator *Ep400* exhibited high expression level in oocytes and 2‐cell embryos, followed by downregulation at later developmental stages. To further investigate the translation activity of *Ep400* mRNA, we analyzed a public Ribo‐seq dataset,^[^ ^24^ ^]^ revealing its consistent translation from oocyte to 2‐cell embryo stage and a gradual reduction in translation activity thereafter (Figure 1c). EP400 is a key component of the TIP60‐EP400 complex, known for catalyzing histone acetylation and chromatin remodeling. Despite the global knockout of *Ep400* in mice causing post‐implantation embryonic lethality,^[^ ^25^ ^]^ its specific roles in oocyte and early embryo development remain unclear.

To investigate the functions of maternal *Ep400* in oocyte and early embryo development, we employed a mating strategy involving *Ep400* ^fl/+^ mice^[^ ^14c^ ^]^ and *Zp3* ^Cre^ transgenic mice to generate cKO female mice with oocyte‐specific depletion of *Ep400* ^[^ ^26^ ^]^ (Figure 1d). The insertion of LoxP cassettes flanking exon 15 allowed for Cre‐mediated disruption, resulting in the loss of ATPase activity and a frameshift mutation in *Ep400*. Genotyping analysis successfully identified cKO mice (Figure S1a, Supporting Information). The knockout of *Ep400* in germinal vesicle (GV) oocytes was confirmed through qRT‐PCR (Figure 1e) and low‐input RNA sequencing (Figure 1f). Due to the absence of a commercially‐available anti‐EP400 antibody for immunofluorescence and the difficulty of western blotting for EP400 with a molecular weight up to 337 kDa (Figure S1b, Supporting Information), we employed a dot blot assay using the anti‐EP400 antibody (Figure S1c, Supporting Information) to validate the depletion of EP400 protein in cKO oocytes (Figure S1d, Supporting Information). A 6‐month fertility test revealed a significant decline in the fertility of female cKO mice, with complete loss of fertility in the cKO occurring around 3 to 4‐month of age (Figure 1g).

### 2.2 Oocyte Loss Occurred in the Ovary of *Ep400* cKO Mice

**Figure 2:**
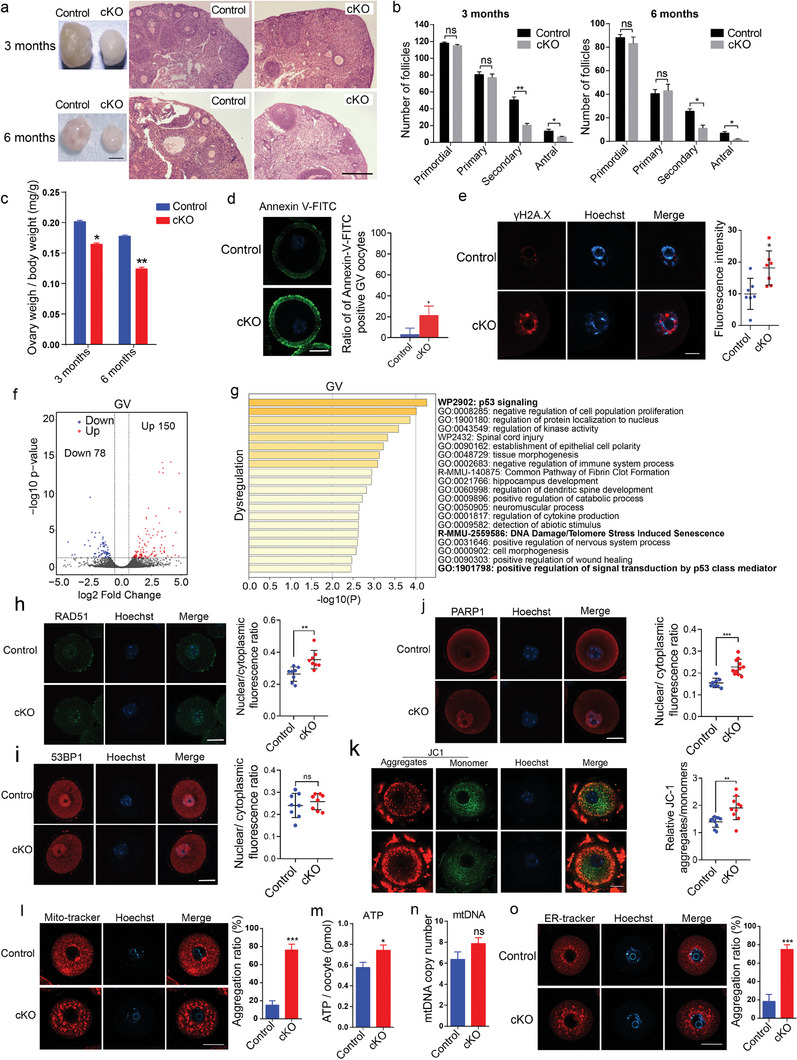
Developmental defect of oocytes from *Ep400* cKO mice. a) Images of ovaries from control and cKO mice at different ages (left), and H&E staining of paraffin‐embedded ovary sections at maximum cross (right). Scale bar, 5 mm (left), 1 mm (right). b) Quantification of mouse ovarian follicles at different developmental stages in ovaries of control and cKO mice. Data are presented as means ± SD (*n* = 3). Two‐tailed student's *t*‐test was used to calculate *p* values. ∗ *p* < 0.05; ∗∗ *p* < 0.01; ns, not significant. c) Ovary weight/body weight ratio of control and cKO mice at different ages. Data are presented as means ± SD (*n* = 3). Two‐tailed student's *t*‐test was used to calculate *p* values. ∗ *p* < 0.05; ∗∗ *p* < 0.01. d) Annexin‐V‐FITC staining of isolated GV oocytes from control and cKO mice for apoptosis examination. Data are presented as means ± SD (*n* = 3). Two‐tailed student's *t*‐test was used to calculate p values. Scale bar, 25 µm. ∗ *p* < 0.05. e) γH2A.X staining of isolated GV oocytes from control and cKO mice. Data are presented as means ± SD (*n* = 7). Two‐tailed student's *t*‐test was used to calculate *p* values. Scale bar, 25 µm. ∗ *p* < 0.05. f) Scatter plot comparing transcripts of isolated GV oocytes between control and cKO group. Only genes with adjusted p value less than 0.05 were regarded as dysregulated. Gene expression with log_2_ (fold change) increased or decreased more than 1 in cKO group is highlighted with red and blue, respectively. g) Gene ontology analysis of dysregulated genes in *Ep400*‐depleted GV oocytes by Metascape. h) RAD51 staining of P17 GO from control and cKO mice. Data are presented as means ± SD (*n* = 8). Two‐tailed student's *t*‐test was used to calculate *p* values. Scale bar, 25 µm. ∗∗ *p* < 0.01. i) 53BP1 staining of P17 GO from control and cKO mice. Data are presented as means ± SD (*n* = 8). Two‐tailed student's t‐test was used to calculate *p* values. Scale bar, 25 µm. ns, not significant. j) PARP1 staining of P17 GO from control and cKO mice. Data are presented as means ± SD (*n* = 8). Two‐tailed student's t‐test was used to calculate *p* values. Scale bar, 25 µm. ∗∗∗ *p* < 0.001. k) JC1 staining of P17 GO from control and cKO mice to detect MMP. MMP level is indicated by red/green ratio. Data are presented as means ± SD (*n* = 10). Scale bar, 25 µm. ∗∗ *p* < 0.01. l) GV Oocytes were stained with Mito‐Tracker Red to show mitochondrial distribution. Data are presented as means ± SD (*n* = 15). Two‐tailed student's *t*‐test was used to calculate *p* values. Scale bar, 25 µm. ∗∗∗ *p* < 0.001. m) ATP level in control and *Ep400*‐depleted GV oocytes. Data are presented as means ± SD (*n* = 6). Two‐tailed student's *t*‐test was used to calculate *p* values. ∗ *p* < 0.05. n) mtDNA copy number of control and *Ep400*‐depleted GV oocytes. Data are presented as means ± SD (*n* = 5). Two‐tailed student's *t*‐test was used to calculate *p* values. ns, not significant. o) Oocytes were stained with ER‐Tracker Red to show ER distribution. Data are presented as means ± SD (*n* = 15). Two‐tailed student's *t*‐test was used to calculate *p* values. Scale bar, 25 µm. ∗∗∗ *p* < 0.001.

Due to stress from internal and external factors, ovarian follicles faced an increased risk of DSBs with advancing age. The prompt repair of these DSBs during oocyte development holds significant importance in safeguarding oocyte quality and preserving female fertility. *Ep400* plays a crucial role in promoting DSB repair through homologous recombination, thereby shielding cells from premature senescence, cell cycle arrest, and apoptosis across various cell types.^[^ ^10^, ^13^, ^14^ ^]^ To explore the impact of *Ep400* on oocyte development with age, we collected ovaries at different stages for HE staining and follicle quantification. Notably, the size and maximum cross section of ovaries from 3 and 6‐month‐old female were markedly smaller in cKO mice (**Figure** **2**a). Corresponding to the age‐related changes in ovarian size, a substantial loss of secondary and antral follicles was observed in 3 and 6‐month cKO mice (Figure 2b). Additionally, the ovary weight/body weight ratio exhibited a significant decrease with age (Figure 2c). In our pursuit of understanding the mechanism underlying *Ep400*’s regulation of oocyte development, we focused on 3‐month aged cKO mice for further investigation unless stated otherwise. TUNEL assay confirmed an upsurge in apoptosis in ovarian sections from cKO mice (Figure S2a, Supporting Information). The percentage of GV oocytes displaying early apoptosis, as indicated by Annexin‐V staining, was higher in cKO mice, supporting the notion of gradual oocyte loss upon *Ep400* depletion (Figure 2d). Furthermore, the γH2A.X signal of oocytes from cKO mice significantly increased, signifying inefficient DSB repair in GV oocytes (Figure 2e; Figure S2b, Supporting Information). Low‐input RNA sequencing approach revealed minor transcriptome disturbance with only 150 upregulated and 78 downregulated genes in GV oocytes from cKO mice (Figure 2f; Figure S2c, Supporting Information). We observed an enrichment of dysregulated genes associated with apoptosis and senescence, as indicated by gene ontology terms such as “p53 signaling”, “DNA damage/telomere stress induced senescence”, and “positive regulation of signal transduction by p53 class mediator” (Figure 2g), and dysregulation of p53 signaling pathway genes was verified by qRT‐PCR (Figure S2d, Supporting Information).

We further investigated RAD51 and 53BP1 foci at P17 growing oocyte (GO) stage since these proteins play pivotal roles in DNA damage repair (Figure 2h,i). The findings revealed an increase in the RAD51 signal, while the 53BP1 signal remained unaltered in cKO oocytes. In response to genomic instability stress, PARP1 activation occurs, leading to mitochondrial fusion and enhanced ATP synthesis, supporting the energy requirements for DSB repair.^[^ ^27^ ^]^ Prolonged or severe DNA damage, if not promptly repaired, results in mitochondrial dysfunction and disrupted energy homeostasis.^[^ ^28^ ^]^ Activation of PARP1, as indicated by PARP1 staining in P17 GO (Figure 2j), and heightened mitochondrial activity, observed through mitochondrial membrane potential (MMP) examination (Figure 2k), were evident. Subsequently, we investigated the distribution and function of mitochondria in grown GV oocytes from adult mice. Abnormal aggregation of mitochondria and increased ATP content were observed in grown GV oocytes from cKO mice (Figure 2l,m; Figure S2e, Supporting Information). However, there were no discernible changes in mtDNA copy number or MMP (Figure 2n; Figure S2f, Supporting Information), suggesting a relatively balanced mitochondrial homeostasis in GV oocytes from cKO mice. The endoplasmic reticulum (ER) forms stable contacts with various organelles, including mitochondria, Golgi apparatus, and lysosomes. Junctions between ER and mitochondria, known for their stability even during cytoskeleton movement,^[^ ^29^ ^]^ exhibited abnormalities along with mitochondria in GV oocytes from cKO mice (Figure 2o). Notably, no irregularities in the distribution of the Golgi apparatus or lysosomes were observed in GV oocytes from cKO mice (Figure S2g,h, Supporting Information). In summary, the deletion of *Ep400* in oocytes resulted in oocytes loss, which can be potentially attributed to inefficient DSB repair and abnormalities in the distribution and function of cytoplasmic organelles.

### 2.3 Maturation Defect and Quality Decline of Oocytes from *Ep400* cKO Mice

**Figure 3:**
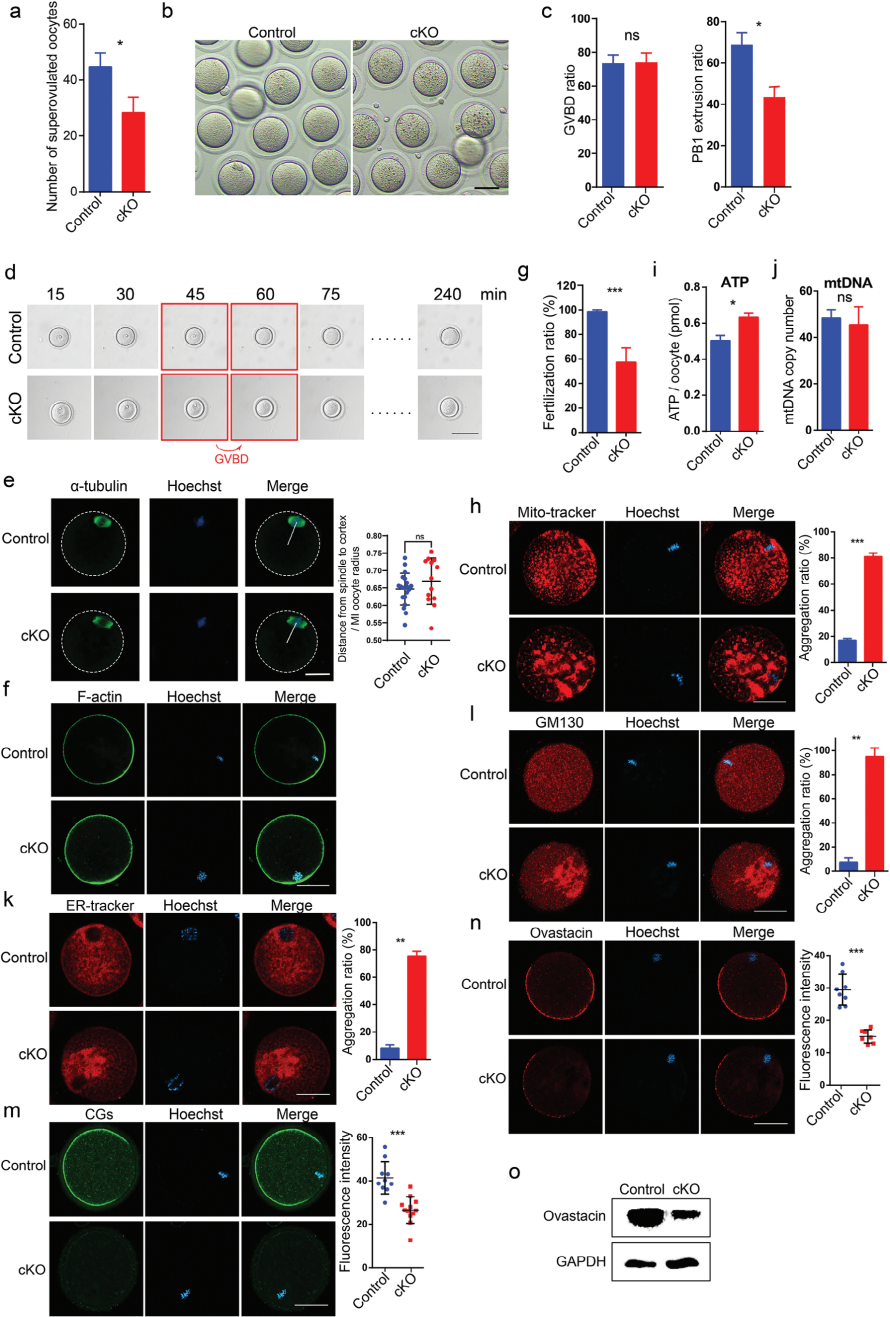
Maturation defect and quality decline of *Ep400*‐depleted oocytes. a) Superovulation of 3‐month‐old control and cKO mice. Data are presented as means ± SD (*n* = 3). Two‐tailed student's *t*‐test was used to calculate *p* values. ∗ *p* < 0.05. b) Morphology of superovulated oocytes from control and cKO mice. Scale bar, 25 µm. c) The ratio of GVBD and PB1 extrusion of oocytes from control and cKO mice after in vitro maturation for 10 h and 18 h, respectively. Data are presented as means ± SD (*n* = 76). Two‐tailed student's *t*‐test was used to calculate *p* values. ∗ *p* < 0.05; ns, not significant. d) Imaging of GV oocytes from control and cKO mice during the in vitro maturation at 15‐minute interval by real‐time image acquisition system. The images enclosed within the red frame depict the time interval when GVBD occurs. Scale bar, 100 µm. e) α‐tubulin staining to show the spindle morphology of oocytes from control and cKO mice. Data are presented as means ± SD (*n* = 18). Scale bar, 25 µm.Two‐tailed student's *t*‐test was used to calculate *p* values. ns, not significant. f) F‐actin staining to show cytoskeleton of oocytes from control and cKO mice. Scale bar, 25 µm. g) Fertilization ratio (in vivo) of oocytes (fertilized oocytes versus total oocytes collected from ampulla of oviduct) from control and cKO mice. Data are presented as means ± SD (*n* = 127). Two‐tailed student's *t*‐test was used to calculate *p* values. ∗∗∗ *p* < 0.001. h) MII oocytes were stained with Mito‐Tracker Red to show mitochondrial distribution. Data are presented as means ± SD (*n* = 15). Two‐tailed student's *t*‐test was used to calculate *p* values. Scale bar, 25 µm. ∗∗∗ *p* < 0.001. i) ATP level in control and *Ep400*‐depleted MII oocytes. Data are presented as means ± SD (*n* = 6). Two‐tailed student's t‐test was used to calculate *p* values. ∗ *p* < 0.05. j) mtDNA copy number of control and *Ep400*‐depleted MII oocytes. Data are presented as means ± SD (*n* = 5). Two‐tailed student's *t*‐test was used to calculate *p* values. ns, not significant. k) MII oocytes were stained with ER‐Tracker Red to show ER distribution. Data are presented as means ± SD (*n* = 15). Two‐tailed student's *t*‐test was used to calculate *p* values. Scale bar, 25 µm. ∗∗ *p* < 0.01. l) MII oocytes were used to stain GM130 to show Golgi apparatus. Data are presented as means ± SD (*n* = 15). Two‐tailed student's *t*‐test was used to calculate *p* values. Scale bar, 25 µm. ∗∗ *p* < 0.01. m) MII oocytes were stained with LCA‐FITC to show CGs. Data are presented as means ± SD (*n* = 12). Two‐tailed student's *t*‐test was used to calculate *p* values. Scale bar, 25 µm. ∗∗∗ *p* < 0.001. n) Ovastacin staining in control and *Ep400*‐depleted MII oocytes. Data are presented as means ± SD (*n* = 8). Two‐tailed student's *t*‐test was used to calculate *p* values. Scale bar, 25 µm. ∗∗∗ *p* < 0.001. o) Western blot analysis of Ovastacin protein in control and *Ep400*‐depleted MII oocytes. 70 control or cKO oocytes were used for each Western blot assay.

Meiotic disorders often arise from abnormalities in DSB repair and cytoplasmic organelles. To further understand this, we explored oocyte maturation in 3‐month‐old cKO mice. Superovulation of female mice led to a significant reduction in mature MII oocytes (**Figure** **3**a). Additionally, MII oocytes from cKO mice displayed brown aggregates in the cytoplasm, as depicted in Figure 3b. The in vitro maturation (IVM) assay highlighted a notable decrease in the rate of first polar body extrusion in oocytes from cKO mice. However, the germinal vesicle breakdown (GVBD) ratio and timing remained normal (Figure 3c,d). Investigation of mitochondria localization in the MI stage revealed increased aggregation in the cytoplasm and reduced proximity to chromosomes in cKO oocytes, suggesting potential inefficiency in providing local energy support for chromosomes and other subcellular organelles (Figure S3a, Supporting Information). Meiotic defect in oocytes is often attributed to spindle abnormalities, with F‐actin playing a crucial role in spindle migration and the extrusion of the first polar body.^[^ ^30^ ^]^ α‐tubulin staining at 8 h post IVM indicated normal spindle morphology and appropriate distance between the spindle and oocyte center in cKO oocytes (Figure 3e). Further F‐actin staining revealed a normal cytoskeleton in oocytes from cKO mice (Figure 3f). These findings suggest that meiosis defect in *Ep400*‐deficient oocytes is not linked to spindle or cytoskeleton abnormalities. The fertilization ratio of *Ep400*‐deficient oocytes was observed to be lower than that of controls, supporting the notion of decreased oocyte quality (Figure 3g; Figure S3b, Supporting Information).

Mitochondria undergo dynamic changes in number and distribution to meet the energy demands of meiosis during oocyte maturation. Initially, mitochondria aggregate around the germinal vesicle before GVBD. Subsequently, they disperse from the perinuclear region and fragment into smaller clusters throughout the cytoplasm post GVBD. Similar dynamic rearrangements occur in the ER and Golgi apparatus during oocyte maturation.^[^ ^31^ ^]^ Consequently, we conducted further examinations into the distribution and function of cytoplasmic organelles in MII oocytes. Significantly larger clusters of mitochondria were observed, and elevated ATP content and MMP were detected in MII oocytes from cKO mice. These findings indicate unbalanced mitochondrial homeostasis during oocyte maturation (Figure 3h,i; Figure S3c,d, Supporting Information). No discernible change in mtDNA copy number was noted in MII oocytes from cKO mice (Figure 3j). In line with mitochondrial dysfunction, abnormal aggregation of the ER and Golgi apparatus was identified, and the fluorescence intensity of lysosomes increased in MII oocytes from cKO mice (Figure 3k,l; Figure S3e, Supporting Information).

Furthermore, we observed a significant increase in the sperm binding ability to the zona pellucida of 2‐cell embryos from cKO mice (Figure S3f, Supporting Information), suggesting an impaired zona pellucida response after fertilization. Cortical granules (CGs) are typically released into the perivitelline space, initiating the zona pellucida reaction to prevent polyspermy following fertilization.^[^ ^32^ ^]^ Ovastacin, encoded by *Astl*, serves as a crucial cortical granule protein that cleaves ZP2 in the zona pellucida, preventing polyspermy.^[^ ^33^ ^]^ Despite the comparable expression level of *Astl* in *Ep400* deleted oocytes and controls (Figure S3g, Supporting Information), our examination revealed a severe loss of both CGs and Ovastacin protein signals in *Ep400*‐depleted MII oocytes (Figure 3m,n). This significant reduction in Ovastacin protein was confirmed by Western blotting (Figure 3o). To explore the possibility that *Ep400* depletion led to abnormal synthesis of Ovastacin and other CG components, we investigated Ovastacin and CGs in GV oocytes through immunofluorescence. Notably, there was a marked loss of both CGs and Ovastacin protein in *Ep400*‐depleted GV oocytes (Figure S3h,i, Supporting Information). This suggests that the synthesis of CG components was hindered in GV oocytes, potentially due to abnormal mitochondrial and ER activity during oocyte development.

Moreover, low‐input RNA sequencing approach revealed minor transcriptome disturbance with only 133 upregulated and 102 downregulated genes in MII oocytes from cKO mice (Figure S4a,b, Supporting Information). Notably, there was an enrichment of upregulated genes associated with apoptosis, as indicated by gene ontology terms such as “positive regulation of endothelial cell apoptotic process” (Figure S4c, Supporting Information).

In summary, our findings suggest that the primary contributors to the maturation defect and diminished quality of oocytes in cKO mice are likely to be exacerbated remodeling disorder and dysfunction of cytoplasmic organelles.

### 2.4 Developmental Arrest of Maternal *Ep400*‐Deficient Early Embryos

**Figure 4:**
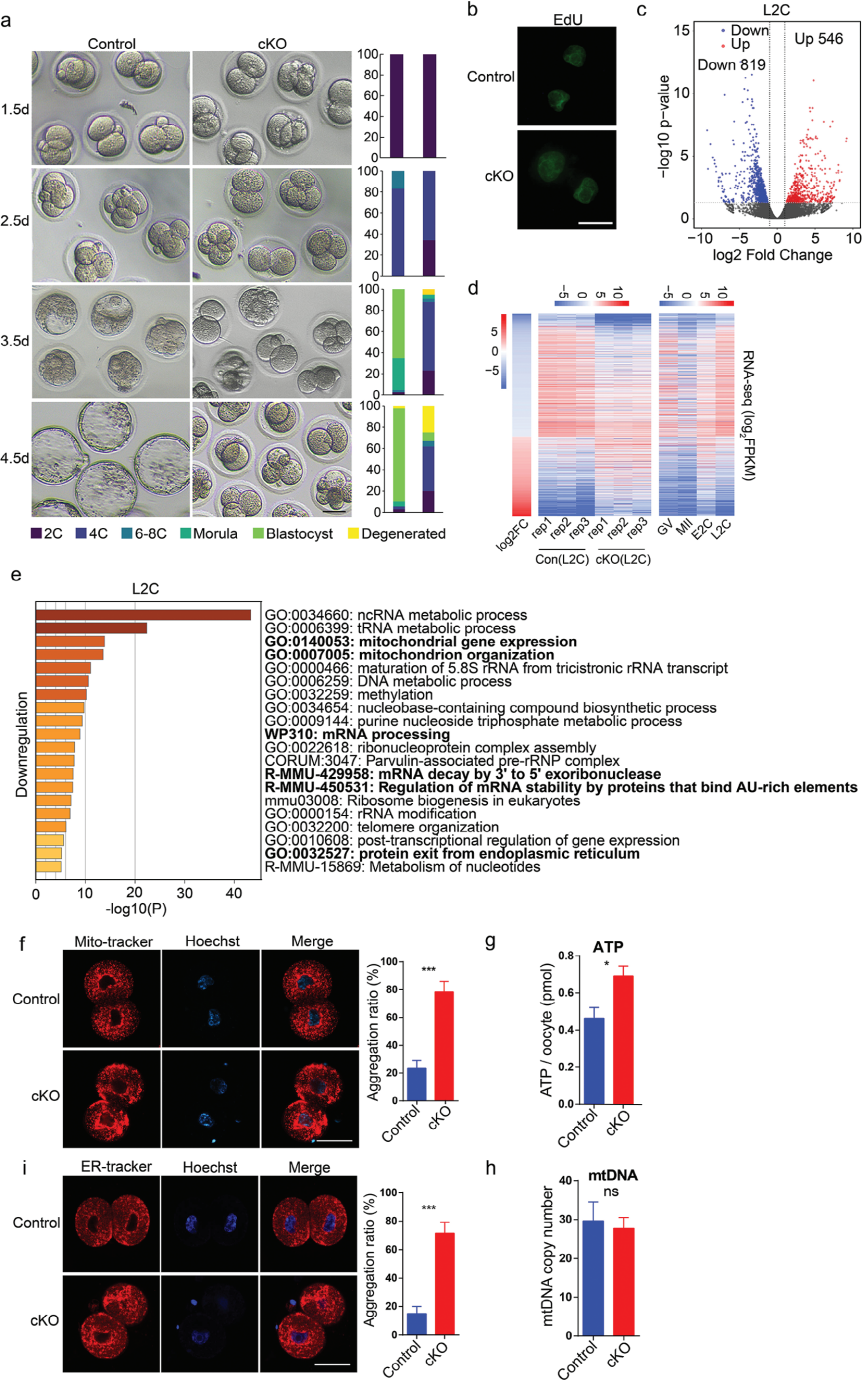
Developmental arrest of maternal *Ep400*‐depleted preimplantation embryos. a) Representative images of in vitro cultured embryos obtained from control and cKO mice mated with WT adult males (left), and corresponding quantification of embryos at different developmental stages (right). Scale bar, 25 µm. b) EdU incorporation assay to detect DNA replication of 2‐cell embryos from control and cKO mice. Scale bar, 100 µm. c) Scatter plot comparing transcripts of late 2‐cell embryos between control and cKO group. Only genes with adjusted p value less than 0.05 were regarded as dysregulated. Gene expression with log_2_ (fold change) increased or decreased more than 1 in cKO group is highlighted with red and blue, respectively. d) Heatmap showing differentially‐expressed genes of late 2‐cell embryos between control and cKO group and their average expression levels at different developmental stages. FC, fold change. e) Gene ontology analysis of downregulated genes in late 2‐cell embryos from cKO mice by Metascape. f) Mitochondrial distribution in control and maternal *Ep400*‐depleted 2‐cell embryos. Data are presented as means ± SD (n = 15). Two‐tailed student's *t*‐test was used to calculate *p* values. Scale bar, 25 µm. ∗∗∗ *p* < 0.001. g) ATP level in control and maternal *Ep400*‐depleted 2‐cell embryos. Data are presented as means ± SD (*n* = 6). Two‐tailed student's *t*‐test was used to calculate *p* values. ∗ *p* < 0.05. h) mtDNA copy number of control and maternal *Ep400*‐depleted 2‐cell embryos. Data are presented as means ± SD (*n* = 5). Two‐tailed student's t‐test was used to calculate *p* values. ns, not significant. i) ER distribution in control and maternal *Ep400*‐depleted 2‐cell embryos. Data are presented as means ± SD (*n* = 15). Two‐tailed student's *t*‐test was used to calculate *p* values. Scale bar, 25 µm. ∗∗∗ *p* < 0.001.

The decline in oocyte quality is a significant factor contributing to early embryonic developmental arrest. To elucidate the roles of maternal *Ep400* in preimplantation embryo development, we conducted mating experiments with 3‐month‐old superovulated cKO females and wildtype (WT) males, and collected zygotes from plugged females for subsequent in vitro culture. Notably, embryos from cKO mice exhibited a predominant arrest at the 2–4 cell stage, with only ≈8% reaching the blastocyst stage, in contrast to about 90% of embryos from control mice that successfully developed to the blastocyst stage (**Figure** **4**a). DNA replication in 2‐cell embryos from cKO mice appeared normal (Figure 4b), indicating that the developmental block occurred from the late 2‐cell stage. Additionally, the γH2A.X signal in control and cKO 2‐cell embryos were comparable, suggesting the elimination of oocytes with severe DNA damage before fertilization (Figure S5a, Supporting Information). We also conducted mating experiments with control/cKO females without superovulation with WT males, and obtained fertilized eggs for subsequent in vitro culture. Generally, fewer 2‐cell embryos were obtained from cKO females, and a majority of early embryos from cKO females experienced developmental arrest at the 2‐to‐4‐cell stage (Figure S5b, Supporting Information). This observation was consistent with the results from the superovulation experiment.

Low‐input RNA sequencing analysis of early 2‐cell embryos revealed minor disturbance in the transcriptome, with 18 upregulated and 36 downregulated genes upon depletion of maternal *Ep400* (Figure S6a,b, Supporting Information). The expression levels of representative ZGA genes were comparable between the control and cKO group (Figure S6c, Supporting Information). Gene ontology analysis of downregulated genes in early 2‐cell embryos indicated enrichment in genes associated with Golgi organization and regulation of organelle assembly (Figure S6d, Supporting Information). In contrast, late 2‐cell embryos from cKO mice displayed more pronounced changes, with 546 upregulated and 819 downregulated genes (Figure 4c,d). Gene ontology analysis of downregulated genes in late 2‐cell embryos showed enrichment in genes related to mitochondrial function and RNA metabolism (Figure 4e). We also checked commonly dysregulated genes in cKO embryos at early and late 2‐cell stage (Figure S6e, Supporting Information). Although there were few overlapped genes, Timm10, a mitochondrial intermembrane chaperone protein, was downregulated in both stages and may contribute to the phenotype.

In accordance with gene ontology analysis, 2‐cell embryos from cKO mice exhibited abnormal mitochondrial aggregation and increased ATP content (Figure 4f,g). However, there were no evident changes in mtDNA copy number, as well as MMP, in 2‐cell embryos from cKO mice (Figure 4h; Figure S7a, Supporting Information), suggesting a relatively balanced mitochondrial homeostasis. Abnormal aggregation of the ER was also observed in 2‐cell embryos from cKO mice (Figure 4i). No abnormities in the distribution of Golgi apparatus or lysosomes were detected in 2‐cell embryos from cKO mice (Figure S7b,c, Supporting Information). Notably, chromosome bridges and micronuclei were observed in 2‐cell embryos from cKO mice (Figure S7d, Supporting Information). These findings collectively suggest that dysregulated gene expression and abnormal organelle distribution contribute to the developmental block of early embryos deficient in maternal *Ep400*.

### 2.5 EP400 Occupied Active Chromatin Regions in Early Embryos

**Figure 5:**
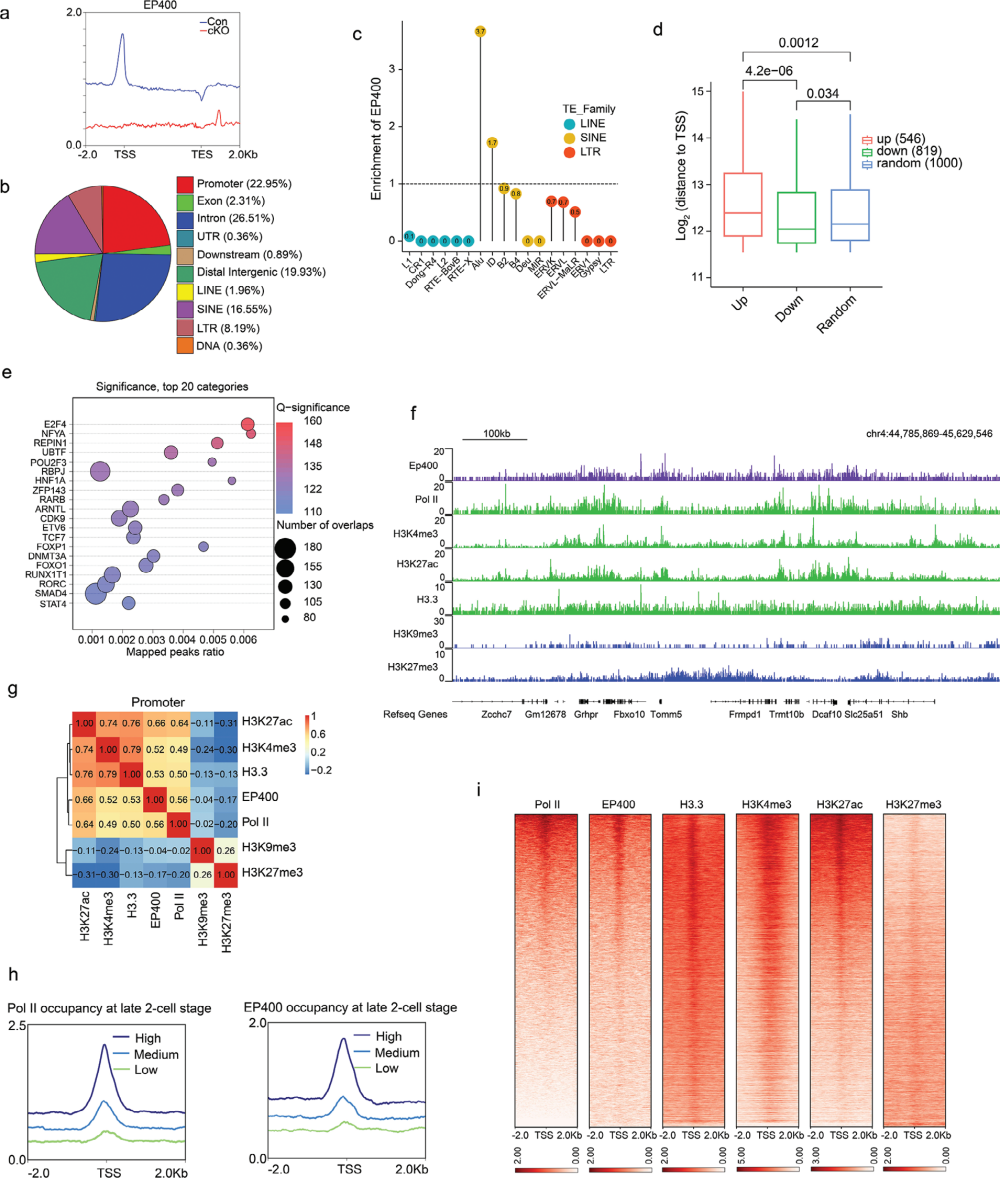
EP400 occupied active chromatin and positively correlated with gene transcription. a) Density plot of EP400 around TSS and 2 kb upstream/downstream of all genes in control and maternal *Ep400‐*depleted late 2‐cell embryos. b) Pie plot showing the percentage distribution of different genomic features including repetitive elements within the EP400‐binding peaks in late 2‐cell embryos. c) Stem bar graph showing the relative enrichment of EP400 on LINE, SINE, and LTR subfamilies, with SINE Alu/B1 being the most enriched family. d) Comparison of distances between TSSs of upregulated/downregulated/randomly‐selected genes (*n* = 546, 819, 1000, respectively) and adjacent distal Alu elements. Mann‐Whitney U test was used to calculate *p* values. e) EP400 binding motifs predicted by EP400‐binding peaks in ReMap database. f) IGV genome browser snapshots of chromatin occupancy of EP400, Pol II, and histone modifications at mouse genome. g) Heatmap representing Pearson correlation of EP400, Pol II, and histone modifications at promoter regions. h) Genes ranked by Pol II enrichment around TSS and 2 kb upstream/downstream of TSS were divided into three groups (upper panel), and average EP400 enrichment around TSS and 2 kb upstream/downstream of these three group genes in WT late 2‐cell embryos (lower panel). i) Heatmap of Pol II, H3.3, and histone modifications around TSS and 2 kb upstream/downstream of all genes ranked by Pol II enrichment in WT late 2‐cell embryos.

To investigate how EP400 regulates gene expression in early embryos, we profiled the genome‐wide distribution profiles of EP400 in late 2‐cell embryos (**Figure** **5**a). The majority of EP400‐bound peaks were located in gene‐rich regions, with 22.95% within gene promoters, 26.51% within gene introns, 2.31% within exons, and 0.36% within UTRs (Figure 5b). Additionally, 19.93% of EP400‐bound peaks were situated in distal intergenic regions. Interestingly, 27.06% of the EP400‐bound peaks were found in transposon regions with SINE class being the most common (16.55%), followed by LTR (8.19%) and LINE classes (1.96%). Analysis of the relative enrichment of EP400 within LINE, SINE, and LTR subfamilies revealed that EP400 binding at SINE B1/Alu subfamily was the most enriched (3.7‐fold enrichment over the entire chromosome), suggesting that SINE B1/Alu elements are the primary retrotransposon targets of EP400 in late 2‐cell embryos (Figure 5c). Given that Alu repeats often serve as active distal regulatory elements, we further examined the distance from TSSs of upregulated and downregulated genes to the nearest EP400‐bound distal Alu elements. The results showed that EP400‐bound Alu elements were much closer to the TSSs of downregulated genes compared to upregulated or randomly selected genes (Figure 5d), implying that EP400 activates gene expression through adjacent Alu elements in late 2‐cell embryos. Motif enrichment analysis of EP400 binding sites identified E2F4, NFYA, and REPIN1 binding sequences as the mostly enriched motifs (Figure 5e). Particularly, NFYA is a crucial maternal factor contributing to DNase I‐hypersensitive site (DHS) formation and ZGA in 2‐cell embryos.^[^ ^34^ ^]^

Additionally, we characterized the genome‐wide distribution profiles of Pol II, H3.3, and histone modifications in late 2‐cell embryos, and compared them with EP400 occupancy (Figure 5f). Pearson correlation analysis revealed a positive correlation between the distribution of EP400 and Pol II on promoters, as well as active histone markers such as H3K4me3 and H3K27ac. In contrast, there was a negative correlation between EP400 distribution with repressive histone markers such as H3K9me3 and H3K27me3 (Figure 5g). To further explore this relationship, we divided all genes evenly into three groups based on the enrichment of Pol II occupancy at their promoter regions, and confirmed that stronger Pol II binding was associated with more robust EP400 binding (Figure 5h). Subsequent analysis of EP400‐associated promoters, sorted by Pol II abundance, confirmed that EP400 primarily bound to actively transcribed genes (Figure 5i). These results strongly suggest that EP400 acts as a positive transcriptional regulator in early embryos.

### 2.6 EP400 Promoted H3.3 Deposition and Transcription Elongation in Early Embryos

**Figure 6:**
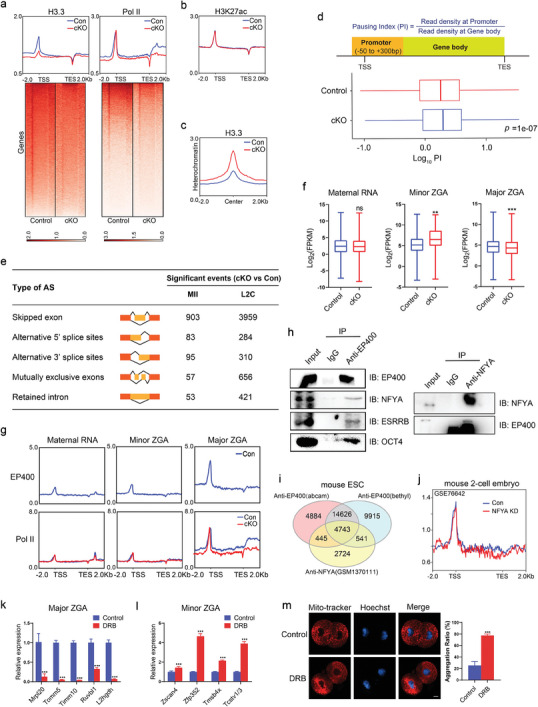
EP400 formed protein complex with transcription factors and promoted transcription elongation. a) Density plot of H3.3 and Pol II around TSS and 2 kb upstream/downstream of all genes in control and maternal *Ep400‐*depleted late 2‐cell embryos (upper panel), and corresponding heatmap (lower panel). b) Density plot of H3K27ac around TSS and 2 kb upstream/downstream of all genes in control and maternal *Ep400‐*depleted late 2‐cell embryos. c) Density plot of H3.3 at 2 kb upstream/downstream of heterochromatin (H3K9me3‐marked regions) center in control and maternal *Ep400‐*depleted late 2‐cell embryos. d) Definition of Pausing Index (PI) and box plot of PI values in control and maternal *Ep400‐*depleted late 2‐cell embryos from Pol II ChIP‐seq data. Mann‐Whitney U test was used to calculate p values. e) Identified significant alternative splicing events (FDR < 0.05) in maternal *Ep400‐*depleted MII oocytes and late 2‐cell embryos. f) Global expression comparison of maternal RNA, minor ZGA, and major ZGA genes between control and maternal *Ep400*‐depleted late 2‐cell embryos by boxplot. Mann‐Whitney U test was used to calculate p values. ns, not significant; ∗∗ *p* < 0.01; ∗∗∗ *p* < 0.001. g) Density plot of EP400 and Pol II around TSS and 2 kb upstream/downstream of maternal RNA, minor ZGA, and major ZGA genes in late 2‐cell embryos. h) co‐IP assay to examine interaction of EP400 with transcription factors NFYA, ESRRB, and OCT4 in mouse ESC. i) Comparison of EP400 peaks (using two different antibodies) and NFYA peaks (GSM1370111) in mouse ESC by Venn diagram. j) Density plot of chromatin opening by low‐input DNase‐seq at loci of downregulated genes induced by maternal *Ep400* deficiency in control and NFYA‐knockdown mouse 2‐cell embryos (GSE76642). k) Relative mRNA level of “major ZGA” genes in late 2‐cell embryos treated with or without DRB by qRT‐PCR. Data are presented as means ± SD (*n* = 3). mRNA levels of genes were normalized to *Actb*. Two‐tailed student's *t*‐test was used to calculate *p* values. ∗∗∗ *p* < 0.001. l) Relative mRNA level of “minor ZGA” genes in late 2‐cell embryos treated with or without DRB by qRT‐PCR. Data are presented as means ± SD (*n* = 3). mRNA levels of genes were normalized to *Actb*. Two‐tailed student's *t*‐test was used to calculate *p* values. ∗∗∗ *p* < 0.001. m) Mito‐tracker staining in late 2‐cell embryos treated with or without DRB to show mitochondrial distribution. Data are presented as means ± SD (*n* = 10). Two‐tailed student's *t*‐test was used to calculate *p* values. Scale bar, 10 µm. ∗∗∗ *p* < 0.001.

To unravel the regulation of chromatin structure by *Ep400* in early embryos, we investigated the distribution of H3.3, Pol II, and histone modifications in late 2‐cell embryos with depletion of maternal *Ep400*. Our findings revealed a substantial negative impact of maternal *Ep400* deficiency on H3.3 and Pol II occupancy on TSS and gene body regions across the mouse genome (**Figure** **6**a). There was no evident change in the distribution of H3K27ac at genic regions upon maternal *Ep400* depletion (Figure 6b). Intriguingly, we observed a significant increase in H3.3 enrichment at heterochromatin regions defined by H3K9me3 peaks, suggesting a redistribution of H3.3 across the mouse genome upon *Ep400* depletion (Figure 6c). Therefore, EP400 appears to exert opposing functions in regulating H3.3 dynamics at euchromatin and heterochromatin in early embryos.

To quantify changes in transcriptional elongation activity, we introduced the Pausing Index (PI) defined as the ratio of accumulated Pol II at the gene promoter region to elongating Pol II at gene body region (Figure 6d). The PI value reflects Pol II pausing in proximity to gene promoters, with a higher PI value indicating less efficient transcription elongation. Our analysis demonstrated a significant increase in the PI value upon *Ep400* depletion, as shown in the boxplot and cumulative curve, signifying less efficient transcription elongation (Figure 6d; Figure S8a, Supporting Information). Given the tight connection between the transcription elongation efficiency and alternative splicing (AS) events due to the transcriptional coupling of these two processes,^[^ ^35^ ^]^ our analysis revealed substantial impacts on AS events in late‐2‐cell embryo stage upon maternal *Ep400* depletion, particularly when compared to the MII stage (Figure 6e). In contrast to reports in somatic cells,^[^ ^9^ ^]^ there was no significant loss of H3.3 occupancy at enhancers, defined as H3K27ac‐occupied intergenic regions. We observed only minor impacts on Pol II and H3K27ac densities at enhancer regions upon depletion of maternal *Ep400*, possibly reflecting unique characteristics of enhancers in early embryos (Figure S8b, Supporting Information).

During mouse cleavage embryogenesis, there is a global degradation of maternal RNA, and a minor transcriptional wave occurs from late 1‐cell to early 2‐cell stages, followed by a major transcriptional wave at the late 2‐cell stage.^[^ ^36^ ^]^ To elucidate how *Ep400* regulates the embryonic transcription program, we compared global abundance of genes representing “maternal RNA”, “minor ZGA”, and “major ZGA” between the control and cKO groups. Gene lists representing these three classes were obtained from a previous report.^[^ ^37^ ^]^ Notably, we observed no change in the abundance of “maternal RNA” abundance, upregulation of “minor ZGA” genes, and downregulation of “major ZGA” (Figure 6f). qRT‐PCR analysis of late 2‐cell embryos confirmed the expression changes in many “minor ZGA” (Figure S9a, Supporting Information) and “major ZGA” (Figure S9b, Supporting Information) genes. Among the differentially‐expressed genes in late 2‐cell embryos, representative “minor ZGA” genes such as *Zscan4* and *Zfp352* were upregulated (Figure S9c, Supporting Information), and increased protein levels of mERVL and ZSCAN4 were identified (Figure S9d,e, Supporting Information). This finding aligns with our analysis that downregulated genes in cKO embryos were those upregulated from early to late 2‐cell stages, and vice versa (Figure 4d). It's worth noting that approximately one third of “minor ZGA” genes were present in the upregulation gene list (Figure S9f, Supporting Information), whereas only ≈10% of “major ZGA” genes were found in downregulation gene list (Figure S9g, Supporting Information). This discrepancy may arise from the utilization of different mouse strains to define “minor/major ZGA”. Importantly, the fold change for the majority of “major ZGA” genes was less than two‐fold, even though they appear predominantly downregulated (Figure S9h, Supporting Information).

In investigating how *Ep400* influenced the three classes of genes in late 2‐cell embryos, we analyzed alterations in chromatin structure. The findings revealed a reduction in Pol II elongation at “major ZGA” genic regions, and no evident change of Pol II distribution at “maternal RNA” or “minor ZGA” genic regions (Figure 6g). Consistent with these results, we observed high abundance of EP400 at “major ZGA” genic regions (Figure 6g).

The upregulation of “minor ZGA” genes and the downregulation of “major ZGA” genes in late 2‐cell cKO embryos raised the possibility of a potential halt in the embryonic transcriptome at the early 2‐cell stage. However, our further in‐depth analysis precludes this possibility. First, DNA replication appeared normal in cKO embryos (Figure 4b). Second, cluster analysis of the transcriptome indicated a higher correlation between late 2‐cell cKO embryo and late 2‐cell control embryos (Figure S9i, Supporting Information). Third, “maternal RNA” in late 2‐cell cKO embryos exhibited more degradation compared to the early 2‐cell stage (Figure S9j, Supporting Information). Finally, we categorized “major ZGA” genes into two classes based on the strength of EP400 binding (Figure S9k, Supporting Information). The analysis revealed that only expression of ZGA genes with strong EP400 binding was significantly downregulated in cKO embryos, while those with weak EP400 binding maintained normal expression levels (Figure S9l,m, Supporting Information). Taken together, these analyses indicated that cKO embryos were not arrested at early 2‐cell stage.

In addressing the inquiry concerning the association of EP400 with the promoters of “major ZGA” genes for activation, we conducted co‐immunoprecipitation (co‐IP) in mouse ESC line AB2.2 (Figure 6h). The interaction between EP400 and NFYA, predicted to be enriched at EP400‐binding sites (Figure 5e), was subsequently confirmed. To validate the potential association of EP400 and NFYA at chromatin, ChIP‐seq was performed using two distinct anti‐EP400 antibodies in mouse ESC. A comparison with publicly available NFYA ChIP‐seq data in mouse ESC revealed significant enrichment of common chromatin regions, supporting EP400‐NFYA interaction at chromatin (Figure 6i). Additionally, we obtained public DNase‐seq data from 2‐cell embryos with/without NFYA knockdown. Our analysis indicated a reduction in DNase‐seq signal at the promoter and gene body regions of downregulated genes induced by maternal *Ep400* depletion (Figure 6j). This finding further supports the co‐occupancy of significant chromatin regions by EP400 and NFYA for gene activation at the mouse genome. Mass spectrometry analysis of the immunoprecipitate identified additional transcription factors and chromatin modifiers (Table S1, Supporting Information). A subsequent co‐IP experiment confirmed the interaction of EP400 with ESRRB and OCT4 proteins (Figure 6h). Consequently, EP400 may form a protein complex with transcription factors to regulate major ZGA. The question then arises how “minor ZGA” genes were upregulated in the absence of *Ep400*. Given that both the upregulation of “major ZGA” genes and the downregulation of “minor ZGA” genes occur at the late 2‐cell stage, it's plausible that the repression of “minor ZGA” was mediated by “major ZGA”. Consequently, mid 2‐cell embryos were treated with 5,6‐dichloro‐1‐β‐d‐ribofuranosyl‐benzimidazole (DRB) to inhibit transcription activity. Late 2‐cell embryos were collected, and qRT‐PCR result revealed efficient inhibition of “major ZGA” gene expression (Figure 6k). Notably, an observed upregulation of “minor ZGA” genes was also confirmed (Figure 6l). Gene ontology analysis of “major ZGA” genes highlighted enrichment in “ribonucleoprotein (RNP) complex biogenesis” (Figure S10a, Supporting Information), suggesting a potential role in controlling the stability of “minor ZGA” transcripts. Additionally, “major ZGA” genes were associated with “mitochondrial organization”, indicating involvement in regulating mitochondrial function. Examination of late 2‐cell embryos treated with DRB revealed severe impairments in mitochondrial distribution (Figure 6m) and MMP (Figure S10b, Supporting Information) in the absence of major ZGA.

Furthermore, we conducted microinjection of siRNA against *Ep400* to assess the functional role of *Ep400* in early embryos. Injection of 3 siRNA sequences into GV oocytes respectively, collection of oocytes 48 h later, and qRT‐PCR analysis of injected oocytes indicated that siRNA‐1 was the most efficient (Figure S11a, Supporting Information). Subsequently, siRNA‐1 was injected into zygotes and embryos were collected at the late 2‐cell stage. The qRT‐PCR analysis revealed the downregulation of *Ep400*, a slight upregulation of “minor ZGA” genes and downregulation of some of “major ZGA” genes (Figure S11b,c,d, Supporting Information). Despite these changes, embryos with *Ep400* knockdown developed normally to the blastocyst stage, potentially attributable to the low efficiency of gene depletion at the early embryo stage (Figure S11e, Supporting Information).

In summary, our findings suggest that EP400 plays a crucial role of mediating H3.3 incorporation at genic regions. This mediation, in turn, facilitates the transcriptional activity of major ZGA which not only represses minor ZGA but also ensures the maintenance of mitochondrial functions at the late 2‐cell stage.

### 2.7 Maternal *Ep400* Depletion Inhibited Tricarboxylic Acid Cycle (TCA Cycle) Enzymes

**Figure 7:**
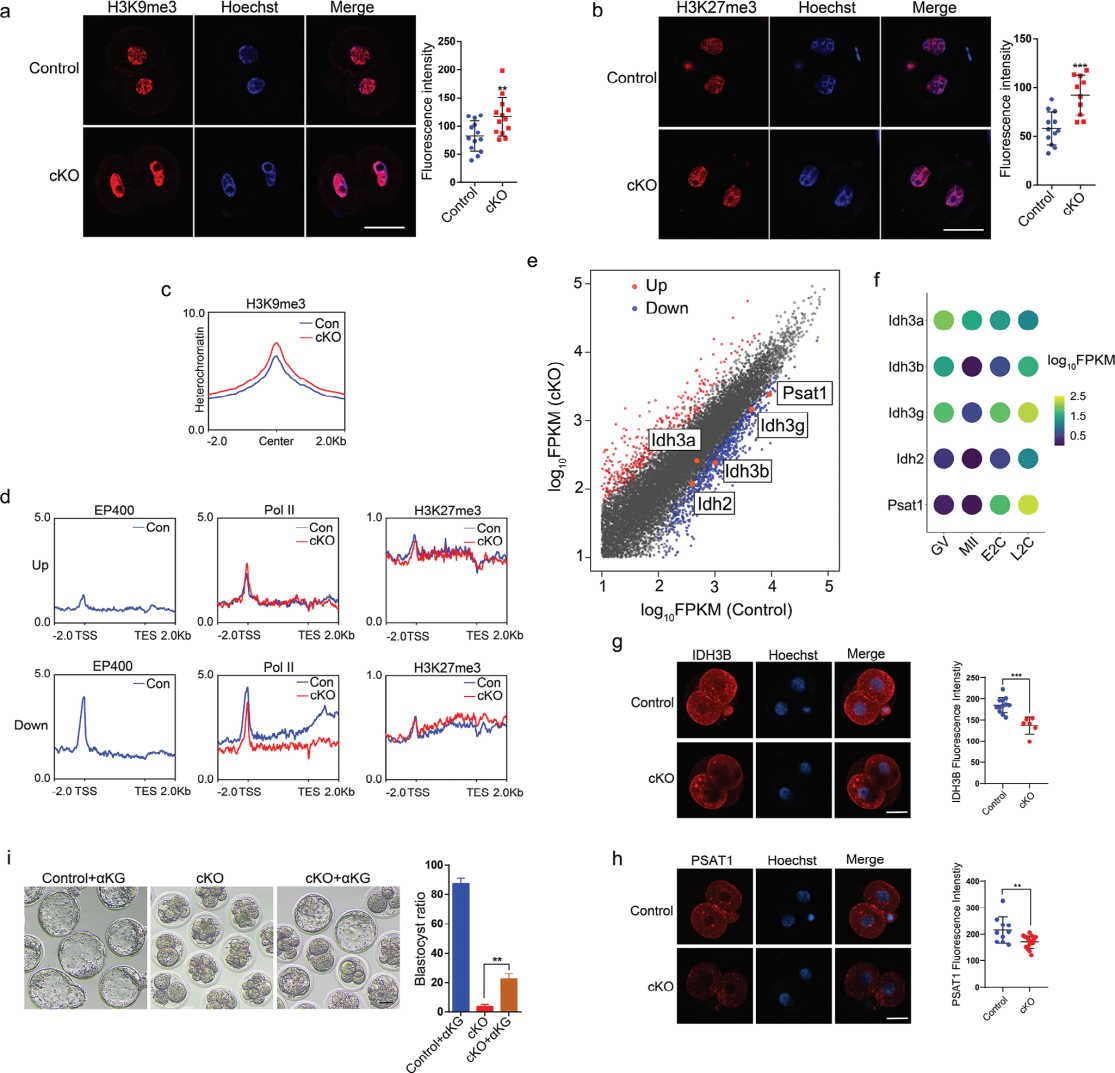
Preimplantation embryo development abnormalities were partially rescued by α‐KG. a,b) H3K9me3 (a) (*n* = 13) and H3K27me3 (b) (*n* = 12) staining in control and maternal *Ep400*‐depleted late 2‐cell embryos. Data are presented as means ± SD. Two‐tailed student's *t*‐test was used to calculate *p* values. Scale bar, 25 µm. ∗∗ *p* < 0.01; ∗∗∗ *p* < 0.001. c) Density plot of H3K9me3 at 2 kb upstream/downstream of heterochromatin (H3K9me3‐marked regions) center in control and maternal *Ep400‐*depleted late 2‐cell embryos. d) Density plot of EP400, Pol II, and histone modification H3K27me3 around TSS and 2 kb upstream/downstream of upregulated (upper panel) and downregulated (lower panel) genes in control and maternal *Ep400*‐depleted late 2‐cell embryos. e) Scatter plot of normalized gene counts in control (x axis) and cKO (y axis) groups with differently expressed genes highlighted. Gene expression with log_2_ (fold change) increased and decreased more than 1 with adjusted P value less than 0.05 is highlighted with red and blue, respectively. Orange‐yellow dots indicate *Idh* family genes and *Psat1* gene. f) Dot plot of expression of *Idh* family genes and *Psat1* gene in mouse GV oocytes, MII oocytes, early 2‐cell embryos, and late 2‐cell embryos. g) IDH3B staining and quantification of control and maternal *Ep400‐*depleted late 2‐cell embryos. Data are presented as means ± SD (*n* = 12). Two‐tailed student's *t*‐test was used to calculate *p* values. Scale bar, 25 µm. ∗∗∗ *p* < 0.001. h) PSAT1 staining and quantification of control and maternal *Ep400‐*depleted late 2‐cell embryos. Data are presented as means ± SD (*n* = 15). Two‐tailed student's t‐test was used to calculate *p* values. Scale bar, 25 µm. ∗∗ *p* < 0.01. i) α‐KG supplementation increased blastocyst ratio of maternal *Ep400*‐depleted embryos, with representative embryo images and quantification of blastocyst ratio shown. Data are presented as means ± SD (*n* = 76). Two‐tailed student's t‐test was used to calculate *p* values. Scale bar, 100 µm. ∗∗ *p* < 0.01.

To investigate whether histone modifications played a role in the regulatory functions of *Ep400*, we examined global changes in several histone marks in late 2‐cell embryos from cKO mice (**Figure** **7**a,b; Figure S12, Supporting Information). Notably, we observed an increase in both H3K9me3 and H3K27me3 intensities in late 2‐cell embryos from cKO mice. Subsequent ChIP‐seq analysis further confirmed the enhanced enrichment of H3K9me3 at H3K9me3‐define heterochromatin regions (Figure 7c). Further investigation into the distribution changes of H3K27me3 and Pol II at genic loci of differentially‐expressed genes revealed increased Pol II occupancy at promoters and decreased Pol II elongation at gene bodies in the loci of upregulated and downregulated genes, respectively. Moreover, EP400 was significantly more enriched at loci of downregulated genes compared to upregulated genes (Figure 7d), consistent with our earlier result indicating that EP400 activates major ZGA through transcriptional elongation (Figure 6g). Importantly, there was an observed increase in H3K27me3 occupancy at loci of downregulated genes (Figure 7d), highlighting the contribution of H3K27me3 to the downregulation of genes in cKO embryos.

Both histone and DNA demethylation processes are facilitated by α‐KG, a cofactor predominantly generated through the oxidative decarboxylation of isocitrate and the oxidative deamination of glutamate. α‐KG serves as a cofactor of JmjC‐domain containing histone demethylases (JHDMs) and DNA demethylases known as ten‐eleven translocation enzymes (Tets)^[^ ^38^ ^]^. Isocitrate dehydrogenase, encoded by the *Idh* gene family, acts as a rate‐limiting enzyme in the TCA cycle, catalyzing the dehydrogenation of isocitrate to produce α‐KG. Additionally, phosphoserine aminotransferase 1 (PSAT1) is a crucial enzyme regulating intracellular α‐KG levels by catalyzing the oxidative deamination of glutamate.^[^ ^39^ ^]^ Our low‐input RNA sequencing results revealed a global decrease in the expression of the *Idh* gene family and *Psat1* in late 2‐cell embryos from cKO mice (Figure 7e). Expression profiles indicated active transcription of *Idh2/3b/3 g* and *Psat1* at the late 2‐cell stage (Figure 7f). Downregulation of IDH3B and PSAT1 at protein level was further confirmed through Immunofluorescence (Figure 7g,h). In subsequent experiments, the exogenous addition of α‐KG to the embryo culture medium during in vitro culture significantly improved the blastocyst formation rate of zygotes from cKO mice and partially inhibited the abnormal increase of repressive histone methylation modifications (Figure 7i; Figure S13, Supporting Information). Furthermore, we knocked down *Ep400* in mouse ESC (Figure S14a, Supporting Information), and ChIP‐qPCR confirmed reduction of H3.3 and Pol II occupancy at *Idh3b* and *Psat1* gene promoters (Figure S14b, Supporting Information).

In summary, our findings suggest that maternal EP400 plays a crucial role in influencing histone modifications, thereby orchestrating the transcriptional program in early embryos through the regulation of mitochondrial function‐related gene expression.

## 3 Discussion

Our research delved into the functions of *Ep400* in mouse oocytes and preimplantation embryos through the utilization of an oocyte‐specific conditional knockout mouse model. Knockout of *Ep400* in mouse oocytes resulted in oocyte developmental and maturation defect, diminished oocyte quality, and abnormalities in fertilization. Maternal depletion of *Ep400* in early embryos led to disruptions in the expression of embryonic genes and aberrant histone modifications, ultimately causing developmental arrest. Mechanistically, our findings revealed that EP400 primarily facilitates DSB repair in oocytes, and promotes transcriptional elongation of major ZGA along with the deposition of H3.3 onto their genic regions in preimplantation embryos. Additionally, EP400 influences histone modifications in 2‐cell embryos by regulating the expression of mitochondrial function‐related genes, such as *Idh* gene family.

### 3.1 Oocyte Development Defect of Conditional Knockout Mice

EP400, a nuclear protein with the ability to catalyze acetylation of histone H2A and H4,^[^ ^10c^ ^]^ is known to regulate gene expression^[^ ^40^ ^]^ and promote DSB repair through homologous recombination.^[^ ^41^ ^]^ Upon *Ep400* knockout, we observed a significant increase in γH2A.X staining in cKO mouse oocytes, indicating aberrant DSB repair. Interestingly, low‐input RNA sequencing revealed minor disturbance in the transcriptome of GV oocytes upon *Ep400* knockout, suggesting that *Ep400* primarily plays a role in DSB repair rather than transcriptional regulation during oocyte development. The initiation of Cre recombinase by the *Zp3* promoter in primary follicles suggests that pre‐transcribed *Ep400* mRNA and protein may partially compensate for the effects of *Ep400* knockout on transcriptional regulation.

We observed abnormal aggregation of mitochondria and ER, along with an increased ATP content, in GV oocytes from cKO mice. Importantly, there was no significant decrease in mtDNA copy number. Our speculation is that the identified abnormal aggregation of mitochondria in oocytes from cKO mice was induced by DSB to generate additional energy for the DSB repair process, without causing damage to mitochondrial structure and function. Moreover, our analysis did not reveal differently‐expressed genes related to organelle distribution and function. Therefore, we conclude that the abnormal distribution of organelles in oocytes from cKO mice is attributed to DSB repair failure, leading to a decline in oocyte quality.

### 3.2 Oocyte Maturation Defect of Conditional Knockout Mice

Oocytes from cKO mice exhibited normal GVBD but displayed partial impairment in the extrusion of the PB1 during in vitro maturation. Furthermore, matured MII oocytes from cKO mice showed the presence of abnormal particulate matter in the cytoplasm. Importantly, spindle morphology and cytoskeleton of oocytes from cKO mice remained normal. However, there was an exacerbation of abnormal aggregation of cytoplasmic organelles such as mitochondria, ER, and the Golgi apparatus. In normal circumstances, ≈95% of mitochondria in the oocyte cytoplasm assemble into clusters with a relatively higher mitochondrial concentration surrounding the spindle. This spatial arrangement enhances ATP synthesis ability, supporting the energy requirements for spindle migration and chromosome separation during meiosis.^[^ ^31a^ ^]^ Oocytes from cKO mice exhibited a more pronounced aggregation of mitochondria compared to controls, along with higher mitochondrial membrane potential and ATP content. However, these large mitochondrial clusters failed to aggregate around the spindle, potentially accounting for the impaired PB1 extrusion of oocytes from cKO mice.

### 3.3 Effect of *Ep400* Knockout on Protein Synthesis in Mouse Oocyte

The observation of a substantial accumulation of sperm in the perivitelline space of unfertilized cKO oocytes raises the possibility that fertilization defect in these oocytes from cKO mice may be attributed to abnormal sperm‐egg recognition and fusion. JUNO and CD9 serve as receptors on the oocyte membrane, mediating sperm‐egg recognition. JUNO recognizes the sperm membrane protein IZUMO1 to facilitate sperm‐egg binding, while CD9 acts as a crucial cofactor in sperm‐egg recognition.^[^ ^42^ ^]^ Notably, our examination indicated that the expression levels of *Juno* and *Cd9* were comparable between oocytes from control and cKO mice. Given that ER and Golgi apparatus are central organelles for the synthesis, processing and transport of secretory proteins, we examined ER and Golgi apparatus and our findings revealed their abnormal aggregation in oocytes from cKO mice. This aberration was accompanied by a significant reduction in the synthesis of the Ovastacin protein and a notable loss of CG, suggesting impaired protein synthesis. Whether the abnormal distribution and function of ER and Golgi apparatus in cKO oocytes lead to synthesis abnormalities of JUNO and CD9 remains a subject for further investigation. Future analyses of the oocyte proteome or protein translatome may provide valuable insights into changes at the protein level following *Ep400* knockout.

### 3.4 Organelle Abnormalities of cKO Mouse Embryos

Abnormal mitochondrial and ER aggregation was also noted in 2‐cell embryos from cKO mice, although the degree of abnormal distribution was less pronounced compared to oocytes from cKO mice. This reduction in abnormal distribution may be attributed to the elimination of oocytes with severe organelle abnormalities through apoptosis during oocyte development and fertilization. In contrast, oocytes with relatively mild organelle abnormalities may survive during oocyte development, maturation, fertilization, and progress to the 2‐cell embryo stage. It is also important to consider the possibility that the embryonic expression of *Ep400* from the paternal allele could potentially contribute to the partial rescue of abnormal organelle distribution at late 2‐cell stage.

### 3.5 Effect of *Ep400* Depletion on Gene Expression in Early Embryos

EP400 distribution across the entire genome was positively correlated with active chromatin markers such as Pol II, and negatively correlated with repressive chromatin markers like H3K27me3 in late 2‐cell embryos. Notably, a higher prevalence of EP400 binding was identified among promoters of downregulated genes compared to upregulated genes in late 2‐cell embryos, implying that EP400 binding at chromatin facilitates gene expression in early embryos. This observation was consistent with the tendency of genes to be downregulated in late 2‐cell embryos upon maternal *Ep400* knockout. In gene ontology analysis of downregulated genes, enrichment was observed in genes associated with mRNA degradation and stability. This was indicated by gene ontology terms including mRNA processing, mRNA decay by 3′ to 5′ exoribonuclease, and regulation of mRNA stability by proteins binding AU‐rich elements. This suggests that the upregulation of these genes may be result from a disequilibrium in RNA metabolism. In mouse ESCs, most differently‐expressed genes were upregulated following *Ep400* knockdown. Despite EP400 being identified to bind to the promoters of 55% of genes and co‐localizing with Pol II and active histone modifications, it may play repressive role in gene expression in ESCs.^[^ ^15^ ^]^ Similarly, knockout of *Ep400* in neural cells predominantly led to gene upregulation.^[^ ^43^ ^]^ These findings collectively suggest that *Ep400* may exert dual regulatory roles in transcription. It is plausible that the upregulation of certain genes in 2‐cell embryos from cKO mice was attributed to the derepression of *Ep400* at their genic loci. Notably, previously reported direct interaction between RAP1 and TIP60‐EP400, where RAP1 enhances the histone acetyltransferase activity of TIP60‐EP400, strengthens the repressive effect of TIP60‐EP400 across a subset of totipotency markers.^[^ ^18b^ ^]^ The question of whether EP400 represses gene expression through its interaction with RAP1 in early embryos remains elusive. Previous reports have highlighted the significance of nucleolar maturation and rRNA synthesis in silencing of totipotent genes and facilitating the exit from the totipotency state in mouse ESC. Suppression of rRNA synthesis has been shown to upregulate *Dux* in mid 2‐cell embyos.^[^ ^44^ ^]^ However, recent research has presented contrasting findings, indicating that a maturated nucleolar structure is essential for transcriptional activation, preventing H3K27me3 of these genes by PRC2 in 2‐cell embryos.^[^ ^45^ ^]^ In our investigation, we observed the downregulation of genes associated with nucleolar function in late 2‐cell embryos of cKO mice, as evidenced by enriched rRNA/ribosome‐related gene ontology terms. The apparent connection between EP400, nucleolar maturation, and the regulation of cleavage embryo genes warrants further exploration and study.

### 3.6 Metabolic Regulation of Histone Methylation in Early Embryos

Gene ontology analysis of downregulated genes in late 2‐cell embryos revealed a significant enrichment of genes associated with mitochondrial function. Subsequent analysis unveiled that these mitochondria‐related genes predominantly encode mitochondrial transmembrane proteins, mitochondrial ribosomal proteins, and tRNA methyltransferase proteins. Additionally, genes involved in TCA cycle and oxidative phosphorylation (OXPHOS) were also downregulated. Beyond their role in energy production, the intermediates of the TCA cycle serve as cofactors for histone and DNA modifying enzymes, orchestrating cellular epigenetic modifications, regulating gene expression, and influencing cell fate.^[^ ^46^ ^]^ The regulatory impact of metabolism on preimplantation embryo development has garnered increasing interest in recent years, with certain metabolic intermediates playing crucial roles in early embryonic development.^[^ ^47^ ^]^ For instance, NAD^+^ has been identified to facilitate the removal of H3K27ac, silencing minor ZGA genes promptly to ensure normal early embryo development.^[^ ^48^ ^]^ In the present study, we observed that *Ep400* potentially influenced histone methylation by regulating α‐KG production in preimplantation embryos. Aside from the *Idh* gene family, we observed a reduction in the expression of other genes associated with the TCA cycle and OXPHOS, including *Sdhb/d*, *Ndufb11b*, and *Acad8*. In mouse ESCs, ESRRB has been identified as an activator of glycolysis‐related genes enhancing OXPHOS activity and facilitating somatic cell reprogramming.^[^ ^49^ ^]^ Interestingly, in early embryos, ESRRB is positioned within TSSs of TCA cycle and OXPHOS genes, exhibiting high intensity for metabolic remodeling and epigenetic reprogramming.^[^ ^50^ ^]^ Mass spectrometry analysis in various cell types has revealed interactions between ESRRB and EP400,^[^ ^51^ ^]^ a finding corroborated by our co‐IP experiment (Figure 6f). Therefore, EP400 may promote the expression of the *Idh* gene family and other TCA cycle/OXPHOS related genes through its interaction with ESRRB in early embryos. However, due to the low sensitivity of current metabolite detection technologies and limitations in experimental materials, direct measurement of metabolic intermediates such as acetyl‐coA, α‐KG, S‐adenosylmethionine (SAM) and others remains challenging. Utilizing low‐input metabolomics assays could provide valuable insights into these aspects. Furthermore, the metabolic regulation of the epigenome by *Ep400* in early embryos warrants further exploration.

## 4 Conclusions

**Figure 8:**
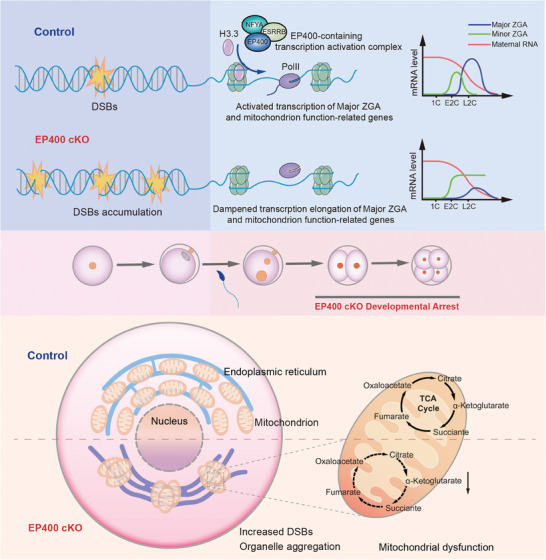
Schematic diagram of the mechanism that *Ep400* regulates oocyte quality and preimplantation embryo development. In oocytes, depletion of *Ep400* caused increased DSBs, which could induce abnormal distribution and dysfunction of cytoplasmic organelles such as mitochondria and ER, causing impaired oocyte development and declined oocyte quality. In preimplantation embryos, maternally‐stored EP400 mainly promoted H3.3 deposition and transcriptional elongation of major ZGA and orchestrated histone modifications, regulating functions of cytoplasmic organelles like mitochondria to ensure developmental program of early embryos.

In summary, our investigation focused on the regulatory roles of *Ep400* in mouse oocyte and early embryo development (**Figure** **8**). Depletion of *Ep400* in oocytes led to accumulated DSBs, causing abnormal aggregation and dysfunction of cytoplasmic organelles, resulting in impaired oocyte development and declined oocyte quality. In preimplantation embryos, EP400 primarily promoted gene transcription and orchestrated histone modifications. Maternal depletion of *Ep400* led to failed major ZGA and abnormalities in cytoplasmic organelles, inducing developmental arrest of early embryos.

## 5 Experimental Section

### Mice

Floxed *Ep400* mice were previously produced as reported.^[^ ^14c^ ^]^ *Zp3* ^Cre^ transgenic mice were bought from Jackson Laboratory and mated with Floxed *Ep400* mice to generate oocyte‐specific conditional knockout mice. Fertility test was performed by mating control/cKO female mice with stud male mice within one cage at a ratio of 1:1. All mice were maintained on C57BL/6J × 129 background.

### Collection and Treatment of Mouse Oocytes and Early Embryos

Adult female mice were injected with 10IU of PMSG followed by 10IU of hCG in 46–48 h to induce superovulation. For oocyte collection, female mice were sacrificed between 46–48 h after PMSG injection. Nude GV oocytes were collected from ovaries which were mechanically cut with ophthalmic scissors in M2 medium (Sigma‐Aldrich, M7167). Similarly, growing oocytes were collected from P17 ovaries for mechanical cutting. For IVM, collected GV oocytes were transferred to equilibrated IVM medium (Nanjing Aibei Biotechnology, M2115) and cultured at 37 °C with 5% CO_2_. MoniCyte^TM^ Intelligent Monitoring System for Living Cells (MC‐F100, Jiangsu Rayme Biotechnology Co., Ltd., China) was used for continuous monitoring with time intervals as 15 min. Cumulus‐oocyte complex (COC) was collected 14 h after hCG injection. MII oocytes denuded of cumulus cells were obtained by hyaluronidase treatment. For IVF, cauda epididymides from adult male mice were lanced in 200 µL of equilibrated HTF medium (Merck, MR‐070‐D) to release sperm, followed by capacitation for 1 h in humidified chamber at 37 °C with 5% CO_2_. Then capacitated sperm were added to COC in 100 µL HTF for 6 h at 37 °C with 5% CO_2_. Successful fertilization was indicated by the presence of two pronuclei. To obtain natural fertilized zygotes, superovulated female mice were caged with stud males in a 1:1 ratio after hCG injection, and successful mating was indicated by the presence of vaginal plugs. Zygotes were collected from ampulla of oviduct and cultured in KSOM medium (Merck, MR‐106‐D) with/without 0.15 mm α‐KG (Sigma‐Aldrich) in a humidified chamber at 37 °C with 5% CO_2_. To inhibit major ZGA, early embryos were treated with DRB as previously reported.^[^ ^52^ ^]^ In brief, DMSO was used to prepare the 200 mm stock solution of DRB (Sigma‐Aldrich, D1916), and 2‐cell embryos were transferred to KSOM medium containing 80 µm DRB after 24 h postinsemination. Control embryos were transferred to DRB‐free medium that contained 0.04% DMSO. After culturing for 9 h, late 2‐cell embryos were collected for further analysis. For siRNA injection, 3 different siRNA sequences (Genepharma, China) against mouse *Ep400* were used: *Ep400*‐targeting siRNA‐1 (antisense sequence: 5′‐UUUGGAGCUGAGUUGAAGCTT‐3′), siRNA‐2 (antisense sequence: 5′‐UUGAAGCUUUGGCAACCGCTT‐3′), siRNA‐3 (antisense sequence: 5′‐AUGACAACAAGAUGAGGGCTT‐3′). The nontargeting siRNA sequence (antisense sequence: 5′‐ACGUGACACGUUCGGAGAATT‐3′; Genepharma, China) was injected as the control. For microinjection of siRNA into mouse oocytes, 5–10 pl of siRNA sequences were injected into cytoplasm of GV oocyte in M2 medium containing 2.5 µm Milrinone using a Narishige microinjector. The working concentration of siRNA was 25 µm. Microinjected oocytes were transferred into IVM medium containing 2.5 µm milrinone and cultured for 48 h, followed by qRT‐PCR for knockdown efficiency examination. For microinjection of siRNA into mouse early embryos, similar parameters were used. Generally, siRNA sequence was injected into cytoplasm of zygotes in M2 medium, microinjected zygotes were then transferred into KSOM for in vitro culture and following examination.

### Cell Culture

The mouse ESC line AB2.2 was cultured on mouse embryonic fibroblast (MEF) feeder cells with ES Cell medium supplemented with 15% FBS (Hyclone, SH30396.03), 1000 U mL^−1^ leukemia inhibitory factor (LIF, Millipore, ESG1107), 3 µm CHIR99021 (LC Laboratories, C‐6556) and 1 µm PD0325901 (LC Laboratories, P‐9688). Cells were cultured in a humidified chamber at 37 °C with 5% CO2 and routinely passaged every other day. Cells were transfected with Lipofectamine 3000 Transfection Reagent (ThermoFisher) according to the manufacturer's instructions.

### RNA Isolation and Quantitative RT‐PCR (qRT‐PCR)

Total RNA was extracted from pooled oocytes (*n* = 50) using TRI reagent (Sigma‐Aldrich, T9424) following the manufacturer's procedure. Reverse transcriptional reaction was performed with Hifair III 1st Strand cDNA Synthesis Kit (Yeasen, 11139ES60) according to the manufacturer's procedure. qRT‐PCR was performed with SYBR green master mix (Yeasen, 11203ES08) on Quantagene q225 qPCR system according to the manufacturers’ instructions. mRNA levels of genes were normalized to *Actb*. For primer sequences, see Table S2 (Supporting Information).

### Histological Analysis and Quantification of Ovarian Follicles

Ovaries were collected and fixed with 4% Paraformaldehyde at 4 °C overnight. Then ovaries were embedded in paraffin. To count the number of follicles, paraffin‐embedded ovaries were serially sectioned at 8 µm from on the maximum cross section and every fifth section was stained with Hematoxylin for morphological observation. Ovarian follicles at different developmental stages, including primordial follicles, Primary follicles, secondary follicles, and antral follicles, were counted in all sections based on the previously established standard.^[^ ^53^ ^]^ In each section, only follicles that contained oocytes with clearly visible nuclei were scored. The total number of follicles at any specific developmental stage was calculated as the sum of the number of follicles from all sections. Images were captured by Olympus BX51 Microscope with MShot MSX2 camera.

### Mitochondria, Mitochondrial Membrane Potential, ER, and Lysosome Detection

For mitochondrion, ER, and lysosome staining, oocytes or embryos were cultured in M2 medium containing cell‐permeant Mito‐Tracker Red CMXRos (1:1000) (Beyotime, C1049B), ER‐Tracker Red (1:600) (Beyotime, C1041S), and Lyso‐Tracker Red (1:1000) (Beyotime, C1046) for 30 min in a humidified chamber at 37 °C with 5% CO_2_ separately. After washing three times with fresh M2 medium for 5 min each time, samples were imaged immediately under the laser scanning confocal microscope (LSM 900, Carl Zeiss). For mitochondrial membrane potential detection, oocytes or embryos were cultured in M2 medium containing 2 µm JC‐1 (Beyotime, C2005) for 30 min in a humidified chamber at 37 °C with 5% CO_2_. After washing three times with fresh M2 medium for 10 min each time, samples were imaged immediately under the laser scanning confocal microscope. Mitochondrial membrane potential was indicated by the ratio of red/green fluorescence intensity.

### Immunofluorescence

Oocytes or embryos were fixed with 3.7% paraformaldehyde containing 0.2% Triton X‐100 at room temperature. Then, samples were blocked with 1% BSA‐supplemented PBS for 1 h and incubated with anti‐γH2A.X antibody (1:100), anti‐GM130 antibody (1:100), anti‐H3.3 antibody (1:100), anti‐H4 antibody (1:100), anti‐H3K4me3 antibody (1:100), anti‐H3K9me3 antibody (1:100), anti‐H3K27me3 antibody (1:100), anti‐mERVL antibody (1:100), anti‐ZSCAN4 antibody (1:100), anti‐α‐tubulin antibody (1:100), LCA‐FITC (1:100), and anti‐Ovastacin antibody (1:100) at 4 °C overnight. After washing with PBST for three times, samples were incubated with Alexa Fluor 594‐conjugated goat anti‐rabbit IgG (H+L) (1:200) or Alexa Fluor 594‐conjugated goat anti‐mouse IgG (H+L) (1:200) for 2 h at room temperature. Finally, samples were counterstained with Hoechst (Sangon Biotech, E607302) for 20 min. Images were obtained using laser scanning confocal microscope. For antibody information, see Table S3 (Supporting Information).

### Western Blot Analysis

Mouse ESCs or oocytes were collected and proteins were extracted using RIPA buffer containing protease inhibitors (Servicebio, G2002, G2006). Equal amounts of protein were separated using SDS‐PAGE gel and then transferred to PVDF membranes. After that, the membranes were blocked in a 5% non‐fat milk solution for 1 h at room temperature and incubated with primary antibodies for 12 h at 4 °C. Following three washes with TBS Tween‐20 solution, the membranes were incubated with a secondary antibody for 2 h at room temperature. Finally, after washing for three times, the membranes were treated with ECL Western Blotting Substrate (Affinity Biosciences, Cat# KF8001) and visualized using ChemiDoc™ XRS+ image system. The expression levels of each genes were normalized to GAPDH as an internal standard.

### Dot Blot Analysis

PVDF membrane with appropriate size was pretreated using methanol, air‐dried properly, then 1–3 µL of cell lysate was spotted onto the PVDF membrane. When the membrane was completely dried, 5% defatted milk was then used for blocking overnight. For subsequent steps, see description in “Western blot analysis”.

### Co‐IP Assay

Mouse ESCs were lysed in 0.5 mL Lysis Buffer with 1% Protease Inhibitor Cocktail. The lysates were incubated with affinity purified anti‐EP400 antibody as the manufacturer's protocol, followed by Western blotting with indicated antibodies (Beyotime, P2177S).

### Measurement of ATP Content

ATP content was measured using ATP determination kit (A22066; Molecular Probes) according to the manufacturer's procedure. Briefly, 20 oocytes or embryos were lysed on ice with 20 µL lysis buffer containing 20 mm Tris (pH7.0), 0.9% Nonidet P‐40, and 0.9% Tween‐20. Standard reaction solution was prepared according to the manufacturer's instructions and placed on ice in the dark before use. 100 µL standard reaction solution was added into each well of 96‐well plates, and then 5 µl samples were added and equilibrated for 1 min before measurement. There were three copies for each sample. A 5‐point standard curve (0, 0.1, 0.5, 1.0,10, and 50 pmol of ATP) was generated in each assay and the ATP content was calculated using the formula derived from the linear regression of the standard curve.

### Mitochondrial DNA (mtDNA) Copy Number Detection

For mtDNA extraction, 20 oocytes or embryos were added to 0.2 mL centrifuge tube containing 20 µL lysis buffer (50 mm Tris‐HCl pH 8.0, 0.5% Triton X‐100, and 200 µg mL^−1^ proteinase K), followed by water bath at 55 °C for 2 h. The relative copy number of mtDNA was estimated by real‐time PCR using ND1 and β‐globin primers. For primer sequences, see primer table (Supporting Information).

### Cell Proliferation Detection

EdU incorporation assay was performed to examine cell proliferation using BeyoClick™ EdU Cell Proliferation Kit with Alexa Fluor 488 (Beyotime, C0071S) according to the manufacturer's procedure. Briefly, embryos 22–24 h post fertilization were cultured in equilibrated KSOM medium containing 10 µm EdU for 5 h. Then, labeled embryos were fixed with 4% paraformaldehyde for 15 min, followed by permeating with 0.3% Triton X‐100 for 15 min. Subsequently, embryos were incubated with click reaction buffer prepared according to the manufacturer's procedure in dark for 30 min at room temperature. Finally, embryos were counterstained with Hoechst (Sangon Biotech, E607302) for 20 min. Fluorescence microscopy (Olympus) was used to obtain images.

### Low‐Input RNA Sequencing Library Construction

10 oocytes or embryos per group were collected in tubes with lysis component and ribonuclease inhibitor. Then the amplification was carried out by the Smart‐Seq2 method. An Oligo‐dT primer was introduced to the reverse transcription reaction for first‐strand cDNA synthesis, followed by PCR amplification to enrich the cDNA and magbeads purification step to clean up the production. Then the cDNA production was checked by Qubit 3.0 Flurometer and Agilent 2100 Bioanalyzer to ensure the expected production with length around 1–2 kbp. Then the cDNA was sheared randomly by ultrasonic waves for Illumina library preparation protocol including DNA fragmentation, end repair, 3′ ends A‐tailing, adapter ligation, PCR amplification, and library validation. After library preparation, PerkinElmer LabChip GX Touch and Step OnePlus Real‐Time PCR System were introduced for library quality inspection. Qualified libraries were then loaded on Illumina Hiseq platform for PE150 sequencing by Annoroad Gene Technology.

### ChIP‐Seq Library Construction

ChIP‐seq was performed using the Hyperactive In‐Situ ChIP Library Prep Kit for Illumina (pG‐Tn5) (Vazyme, TD901) according to the manufacturer's procedure with anti‐EP400 antibody, anti‐H3.3 antibody, anti‐Pol II antibody, anti‐H3K4me3 antibody, anti‐H3K27me3 antibody, anti‐H3K27ac antibody, and anti‐H3K9me3 antibody. A total of 100–200 early embryos were used in each ChIP‐seq experiment. Generally, cells were bound to concanavalin A‐coated magnetic beads for permeabilization, followed by reaction with the antibody and Hyperactive pG‐Transposon adapter complex to cleave the genome near target protein. PCR amplification was performed to generate the library. Qualified libraries were then loaded onto the Illumina Hiseq platform for PE150 sequencing by Annoroad Gene Technology. To validate anti‐EP400 antibodies, two different antibodies against EP400 (from Abcam and Bethyl) were used for ChIP‐seq library preparation with about 5×10^4^ mouse ESC as starting materials. For ChIP‐qPCR validation, SimpleChIP Enzymatic Chromatin IP Kit (Cell Signaling) was used to obtain ChIP and Input samples of mouse ESC, and qPCR was performed with SYBR green master mix on Quantagene q225 qPCR system, with qPCR signals from ChIP samples normalized to that of respective Input. For antibody information, see antibody table of supplementary materials.

### RNA‐Seq Dataset Analysis

Raw reads were processed with cutadapt v1.16 (https://cutadapt.readthedocs.io) to remove adapters and perform quality trimming with default parameters. Trimmed reads were mapped to the mouse genome (GENCODE release M23) using STAR (v2.5.1b) with default settings. Reads were counted in exons of the mouse genome (GENCODE release M23) using the STAR‐quantMode GeneCounts setting. Differential expression of genes for all pairwise comparisons was assessed by DESeq2 v1.24.0 with internal normalization of reads to correct for library size and RNA composition bias. Differentially regulated genes in the DESeq2 analysis were defined as those which were more than two‐fold increased or decreased with adjusted *P* < 0.05. RSEM was used to calculate FPKM value for each gene. Gene ontology analysis was performed by Metascape and enrichplot R package. rMATS (v4.1.1) was used to identify alternative splicing events with “–readLength 150” and other default parameters.

### ChIP‐Seq Dataset Analysis

Raw reads were processed with Trim Galore (v0.6.4) to remove adaptor sequences and poor quality bases with “‐q 20 ‐phred33 ‐stringency 5 ‐length 20 ‐paired.” Trimmed reads were then aligned to the mouse reference genome (mm10) using Bowtie2 (version 2.4.2) with default parameters. Picard (version 2.26.6) was used to remove PCR duplicates. Peaks were called using MACS2 v2.2.7.1 with default parameters. To identify enrichment of transcription factor binding sites, ReMap database (https://remap2022.univ‐amu.fr/) was used. The UCSC Genome Browser utility, bedGraphToBigWig, was used to transform the bedgraph files to bigwig files.^[^ ^54^ ^]^ Tracks were visualized by Integrative Genomics Viewer (IGV). Heatmaps of ChIP‐seq signal enrichment were generated by the Python package, deepTools v3.5.1.^[^ ^55^ ^]^ ChIP‐seq peak annotation was performed by ChIPseeker v1.34.1.^[^ ^56^ ^]^ Intergenic H3K27ac peaks were defined as enhancer regions, and H3K9me3 peaks were defined as heterochromatin regions.

To obtain Pausing Index (PI) value by Pol II ChIP‐seq data, we mapped aligned Pol II ChIP‐seq reads to “Promoter” region (−50 to +300 bp near TSS) and “Gene body” region (+300 bp downstream of TSS to transcription end site (TES)) of individual genes, and calculated the read densities of these regions by deepTools. Then those genes were selected with read density of both “Promoter” and “Gene body” region above 0.1 for subsequent PI value calculation through dividing read density at “Promoter” region by at “Gene body” region.

Enrichment of specific repetitive elements was calculated by the ratio of the length of peaks covering repetitive elements to the length of total peaks versus the ratio of the length of repetitive elements to the chromosome size of the mouse genome. To calculate distance between TSSs and adjacent distal SINE B1/Alu elements, all SINE B1/Alu elements were annotated according to GENCODE annotation (vM23), and obtained distances between each distal SINE B1/Alu element and its adjacent TSS. Only most adjacent SINE B1/Alu elements of each gene were retained as the distance between TSS and SINE B1/Alu.

### Statistical Analysis

Data are presented as means ± SD. Experiments were repeated for at least three times. Two‐tailed student's t‐test by Excel and Mann‐Whitney U test by R were used to calculate *p* values. Statistically significant values for ∗ *p* < 0.05, ∗∗ *p* < 0.01, and ∗∗∗ *p* < 0.001 were indicated by single, double, and triple asterisk, respectively.

### Ethics Statement

Animal procedures were approved by the Institutional Animal Care and Use Committee of Tongji Medical College, Huazhong University of Science and Technology (IACUC Number 3028). Mice were housed in the specific pathogen‐free facility of Huazhong University of Science and Technology. All experiments with mice were conducted ethically according to the Guide for the Care and Use of Laboratory Animal guidelines.

## Conflict of Interest

The authors declare no conflict of interest.

## Author Contributions

Q.T., Y.Y., Y.T., contributed equally to this work. L.Q.Z., X.H., and W.X. conceived, designed, and supervised the study. Q.T. performed the experiments. Y.Y. performed high‐throughput data analysis. Y.T. performed the mouse breeding and helped with the experiments. Y.W. and Y.F.W. helped with the experiments. R.F. and T.F. helped with mouse breeding. A.H.L., L.L., and W.Z. helped with the experimental design and provided valuable guidance. Q.T. wrote the initial manuscript. L.Q.Z. revised the manuscript. All authors contributed to the article and approved the manuscript.

## Supporting information

Supporting Information

## Data Availability

The data that support the findings of this study are openly available in NCBI, reference number PRJNA977124.
