# Supplementary material for: Chromatin Modifier EP400 Regulates Oocyte Quality and Zygotic Genome Activation in Mice
Source: Adv Sci (Weinh). 2024 Mar 17;11(20):2308018. doi: 10.1002/advs.202308018 (PMC11132066; doi:10.1002/advs.202308018)
Supplement: Supplementary file 1 — Supporting Information [file ADVS-11-2308018-s001.pdf]

ADVANCED SCIENCE
Open Access

Supporting Information

for *Adv. Sci.*, DOI 10.1002/advs.202308018

Chromatin Modifier EP400 Regulates Oocyte Quality and Zygotic Genome Activation in Mice

Qing Tian, Ying Yin, Yu Tian, Yufan Wang, Yong-feng Wang, Rikiro Fukunaga, Toshihiro Fujii, Ai-hua Liao, Lei Li, Wei Zhang, Ximiao He, Wenpei Xiang* and Li-quan Zhou**

Chromatin Modifier EP400 Regulates Oocyte Quality and Zygotic Genome Activation in Mice

Qing Tian[1,2]†, Ying Yin[3,4,5,]†, Yu Tian[1,]†, Yufan Wang[1], Yong-feng Wang[1], Rikiro Fukunaga[6], Toshihiro Fujii[6], Ai-hua Liao[1], Lei Li[7], Wei Zhang[2], Ximiao He[3,4,5,*], Wenpei Xiang[1,*], Li-quan Zhou[1,*]

[1]Institute of Reproductive Health, Tongji Medical College, Huazhong University of Science and Technology, Wuhan, Hubei, China

[2]Department of Gynecology and Obstetrics, Zhongnan Hospital of Wuhan University, Wuhan, Hubei, China

[3]Department of Physiology, School of Basic Medicine, Tongji Medical College, Huazhong University of Science and Technology, Wuhan, Hubei, China

[4]Center for Genomics and Proteomics Research, School of Basic Medicine, Tongji Medical College, Huazhong University of Science and Technology, Wuhan, Hubei, China

[5]Hubei Key Laboratory of Drug Target Research and Pharmacodynamic Evaluation, Huazhong University of Science and Technology, Wuhan, Hubei, China

[6]Department of Biochemistry, Osaka Medical and Pharmaceutical University, Takatsuki, Osaka, Japan

[7]State Key Laboratory of Stem Cell and Reproductive Biology, Institute of Zoology, Chinese Academy of Sciences, Beijing, China

†These authors contributed equally

***Correspondences:**

Li-quan Zhou, Tel: +86-27-83692651, Email: zhouliquan@hust.edu.cn

Wenpei Xiang, Tel: +86-27-83692651, Email: wpxiang2010@hust.edu.cn

Ximiao He, Tel: +86-27-83692622, Email: ximiaohe@hust.edu.cn

Supplemental Figures

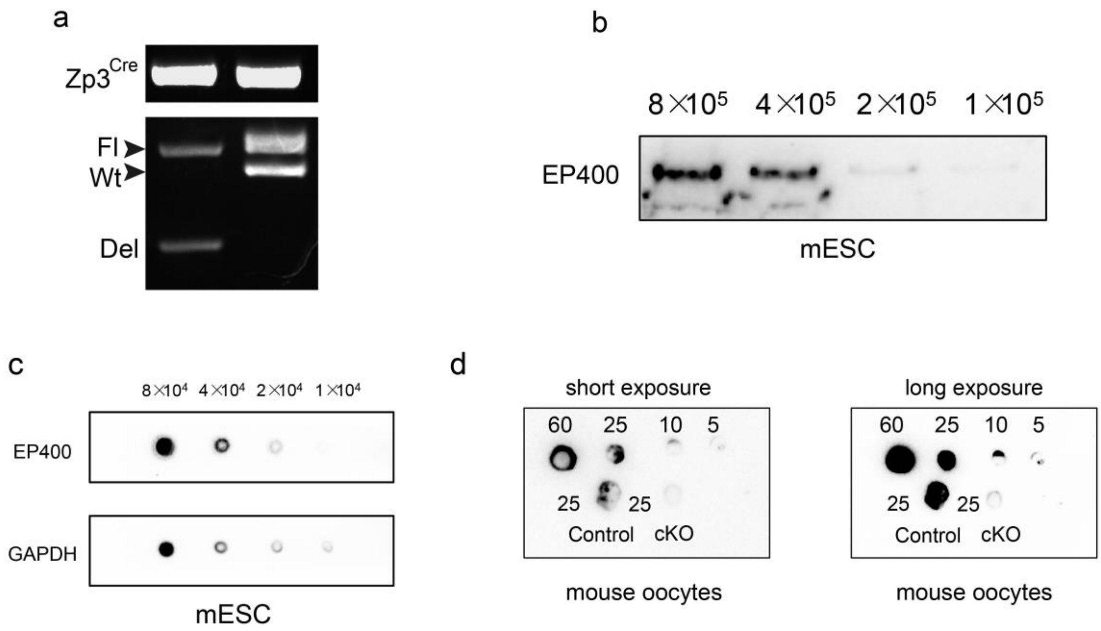

Figure S1. Generation of maternal *Ep400* deficient mice.

(a) Representative genotyping results of $Zp3^{Cre}$, and Fl, Wt, Deleted (Del) alleles.

(b) Western blotting of EP400 using protein extracts from mouse ESC with indicated cell numbers.

(c) Dot blot of EP400 using protein extracts from mouse ESC with indicated cell numbers for imaging assessment.

(d) Dot blot of EP400 using protein extracts from mouse GV oocytes with indicated cell numbers for imaging assessment with short (left) and long (right) exposure.

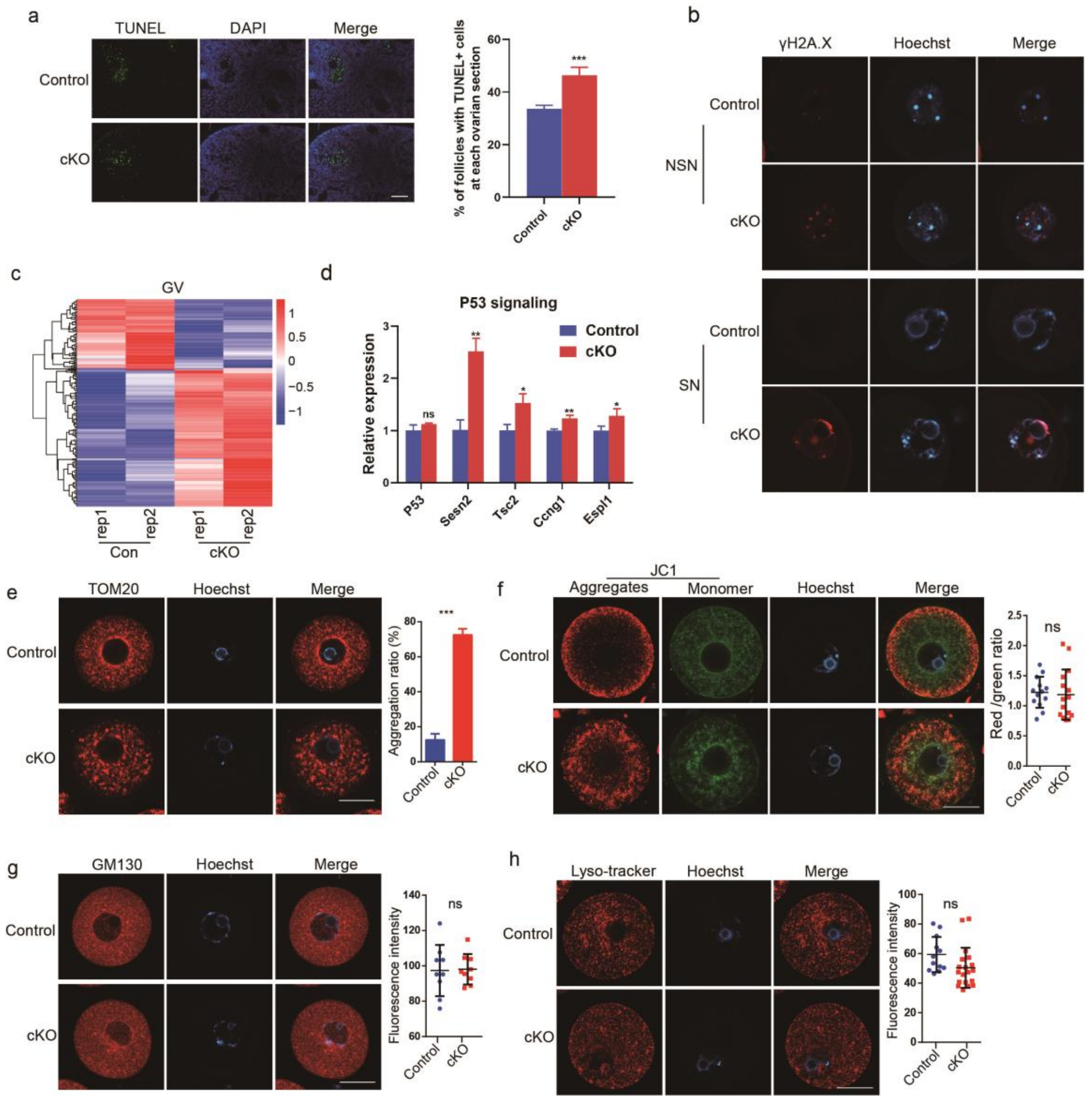

Figure S2. Organelle aggregation of oocytes from cKO mice.

(a) TUNEL staining of ovarian sections from adult control and cKO mice. Data are presented as means ± SD (n=8). Two-tailed student's t-test was used to calculate *p* values. Scale bar, 100μm. $***p < 0.001$.

(b) γH2A.X signal of NSN and SN GV oocytes from control and cKO mice.

(c) Heatmap of differentially expressed genes in GV oocytes between control and cKO group.

(d) Relative mRNA level of "P53 signaling" genes in GV oocyte from control and cKO mice by qRT-PCR. Data are presented as means ± SD (n=3). mRNA levels of genes were normalized to *Actb*. Two-tailed student's t-test was used to calculate p values. ns, not significant, $*p < 0.05$, $**p < 0.01$.

(e) TOM20 staining in control and *Ep400*-depleted GV oocytes to show mitochondrial distribution. Data are presented as means ± SD (n=15). Two-tailed student's t-test was used to calculate *p* values. Scale bar, 25μm. $***\ p < 0.001$.

(f) JC1 staining in control and *Ep400*-depleted GV oocytes to detect MMP. MMP level is indicated by red/green ratio. Data are presented as means ± SD (n=14). Two-tailed student's t-test was used to calculate *p* values. Scale bar, 25μm. ns, not significant.

(g) GM130 staining in control and *Ep400*-depleted GV oocytes to show distribution of Golgi apparatus. Data are presented as means ± SD (n=10). Two-tailed student's t-test was used to calculate *p* values. Scale bar, 25μm. ns, not significant.

(h) GV oocytes were stained with Lyso-Tracker Red to show lysosome distribution. Data are presented as means ± SD (n=12). Two-tailed student's t-test was used to calculate *p* values. Scale bar, 25μm. ns, not significant.

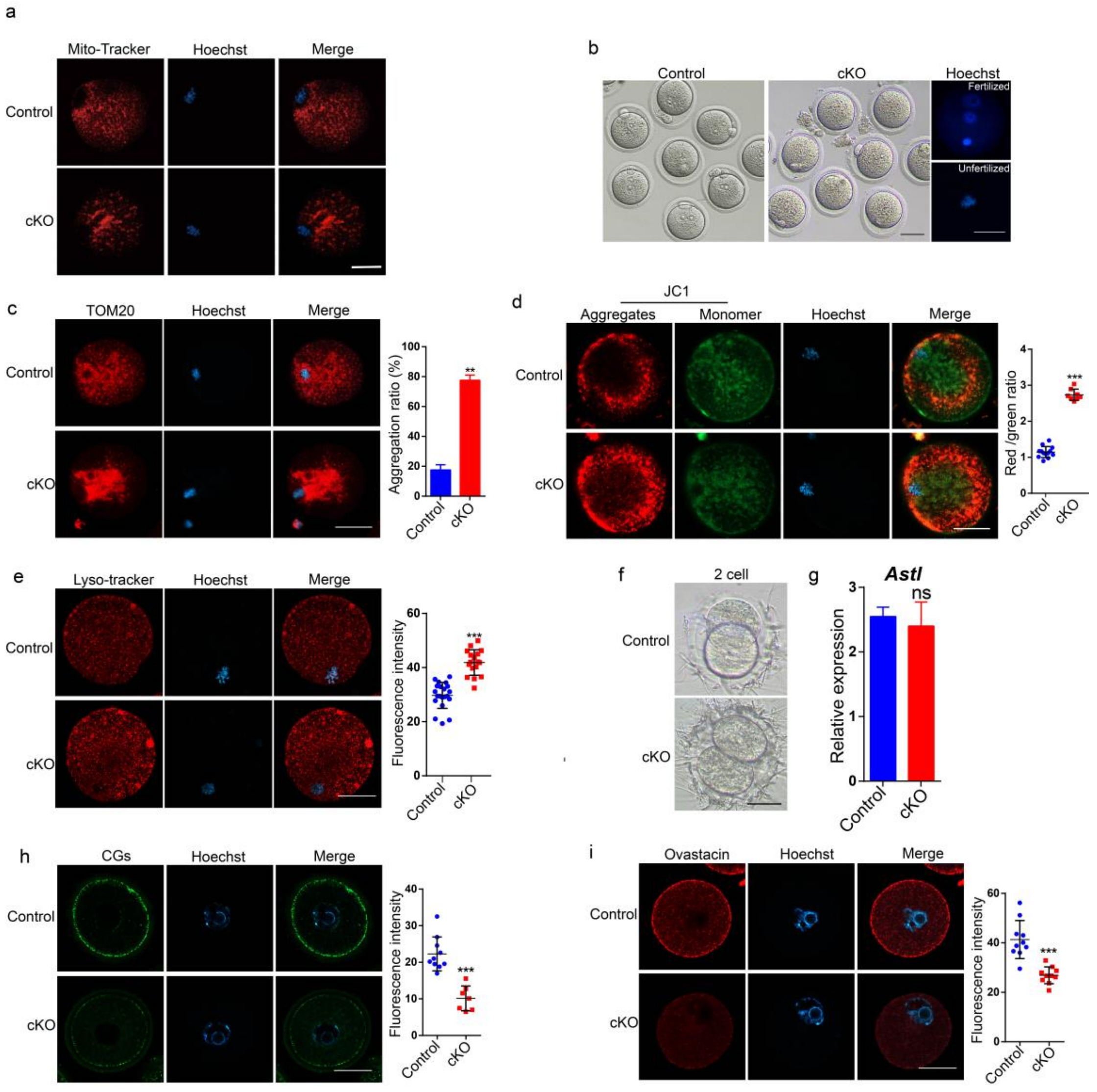

Figure S3. Maturation failure and quality decline of *Ep400*-depleted oocytes.

(a) MI oocytes (8h following IVM) were stained with Mito-Tracker Red to show mitochondrial distribution. Scale bar, 25μm.

(b) Images of fertilized and unfertilized oocytes from control and cKO mice. Scale bar, 50μm.

(c) TOM20 staining in control and *Ep400*-depleted MII oocytes to show mitochondrial distribution. Data are presented as means ± SD (n=15). Two-tailed student's t-test was used to calculate *p* values. Scale bar, 25μm. ** $p < 0.01$.

(d) JC1 staining in control and *Ep400*-depleted MII oocytes to detect MMP. MMP level is indicated by red/green ratio. Data are presented as means ± SD (n=15). Two-tailed student's t-test

was used to calculate *p* values. Scale bar, 25μm. *** $p < 0.001$.

(e) MII oocytes were stained with Lyso-Tracker Red to show lysosome in MII oocytes. Data are presented as means ± SD (n=21). Two-tailed student's t-test was used to calculate *p* values. Scale bar, 25μm. *** $p < 0.001$.

(f) Images of sperm binding to the zona pellucida of control and *Ep400*-depleted 2-cell embryos. Scale bar, 25μm.

(g) Relative expression level of *Astl* encoding ovastacin in MII oocytes from control and cKO mice. Data are presented as means ± SD (n=3). Two-tailed student's t-test was used to calculate *p* values. ns, not significant.

(h) LCA-FITC staining in control and *Ep400*-depleted GV oocytes show CGs. Data are presented as means ± SD (n=10). Two-tailed student's t-test was used to calculate *p* values. Scale bar, 25μm. *** $p < 0.001$.

(i) Ovastacin staining in control and *Ep400*-depleted GV oocytes. Data are presented as means ± SD (n=10). Two-tailed student's t-test was used to calculate *p* values. Scale bar, 25μm. *** $p < 0.001$.

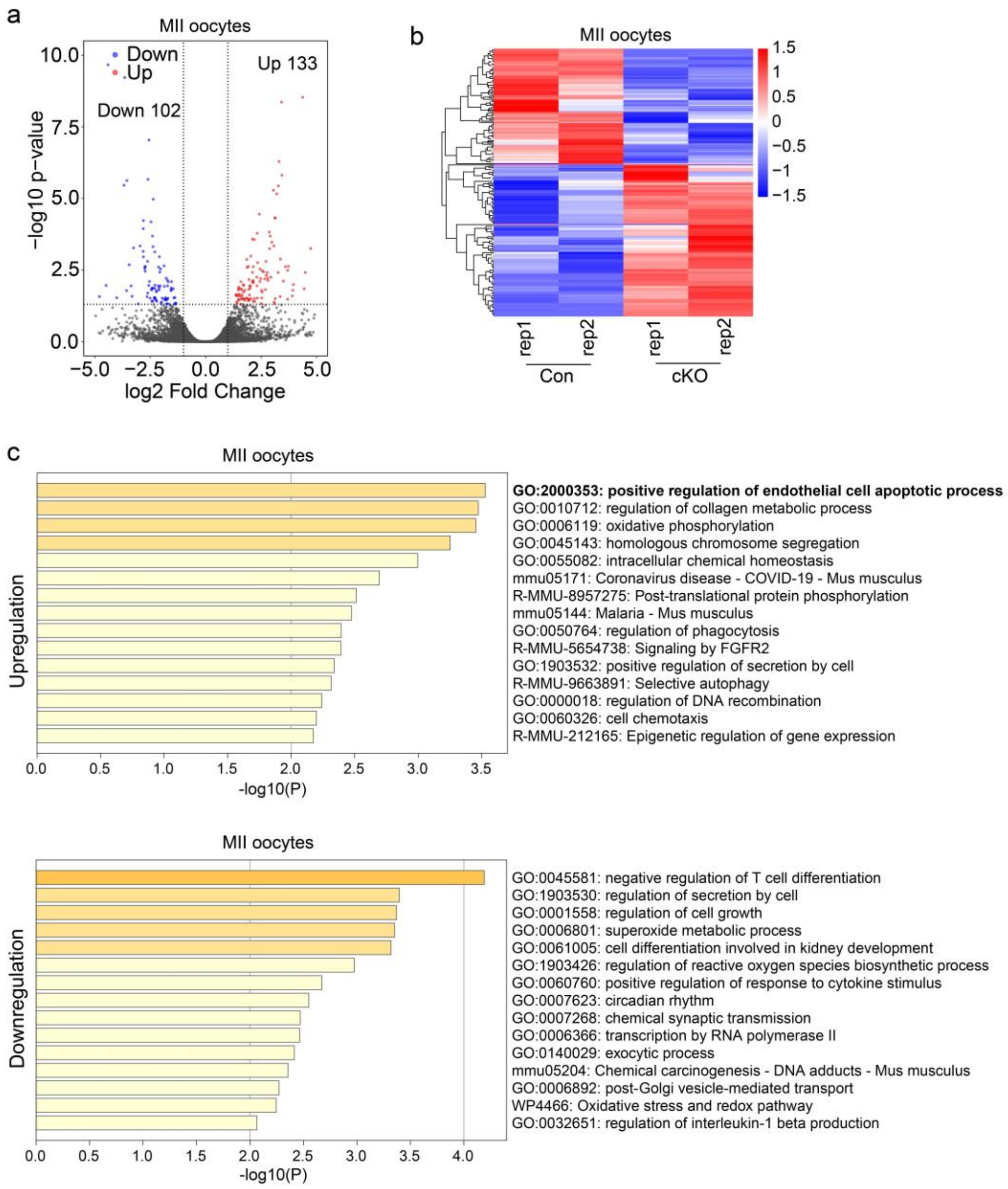

Figure S4. Effect of *Ep400* depletion on transcriptome of MII oocyte.

(a) Volcano plot comparing transcripts of MII oocytes between control and cKO group. Gene expression with $\log_2$ (fold change) increased or decreased more than 1 in cKO group is highlighted with red and blue respectively.

(b) Heatmap of differently expressed genes in MII oocytes between control and cKO group.

(c) Gene ontology analysis of upregulated and downregulated genes in MII oocytes by Metascape.

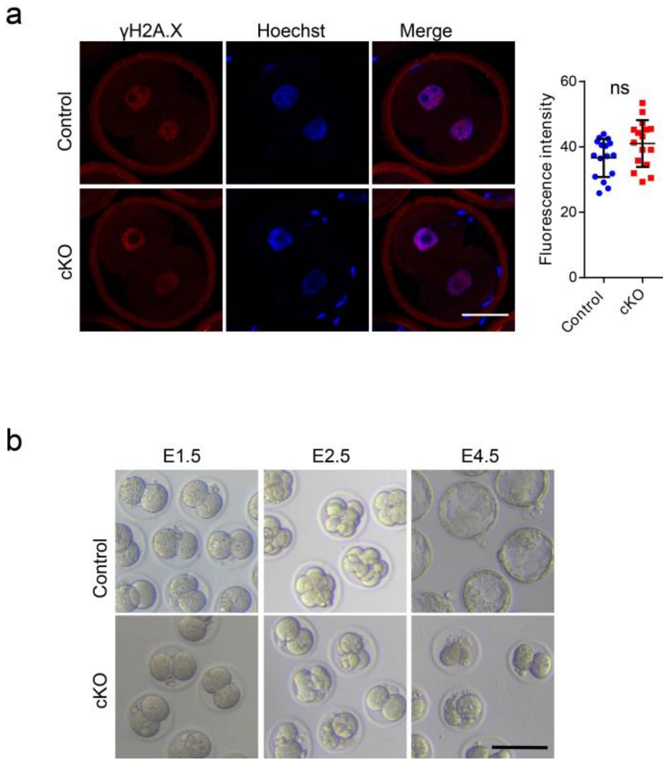

Figure S5. Effect of *Ep400* depletion on early embryos.

(a) Comparison of γH2A.X signal of early 2-cell embryos between control and cKO group. Data are presented as means ± SD (n=16). Scale bar, 25μm. Two-tailed student's t-test was used to calculate *p* values. ns, not significant.

(b) Representative images of embryos obtained from natural cycle ovulation of control and cKO female mated with WT male mice and cultured in KSOM medium. Scale bar, 100μm.

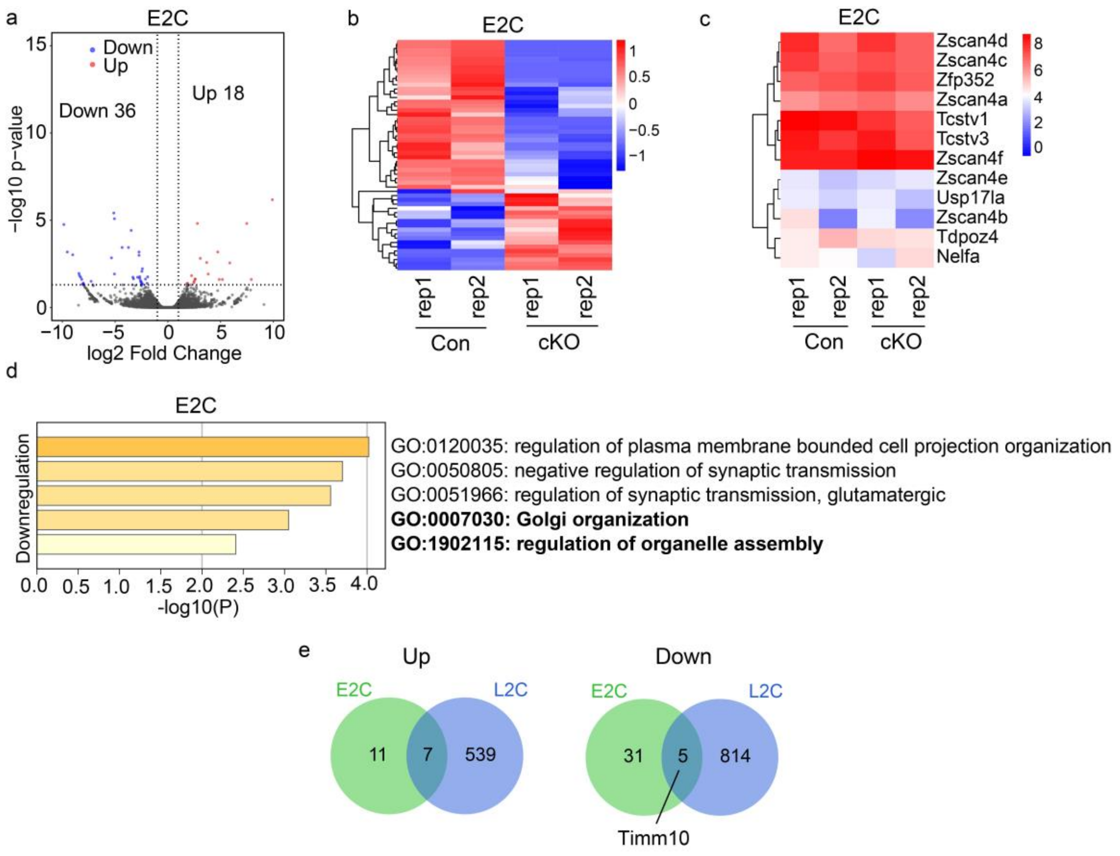

Figure S6. Effect of *Ep400* depletion on transcriptome of early 2-cell embryos.

(a) Volcano plot comparing transcripts of early 2-cell embryos between control and cKO group. Gene expression with $\log_2$ (fold change) increased or decreased more than 1 in cKO group is highlighted with red and blue respectively. E2C, early 2-cell embryos.

(b) Heatmap of differently expressed genes in early 2-cell embryos between control and cKO group.

(c) Heatmap of expression ($\log_2$FPKM) of representative ZGA genes in early 2-cell embryos.

(d) Gene ontology analysis of downregulated genes in early 2-cell embryos by Metascape.

(e) Overlapping of dysregulated genes at early and late 2-cell stages.

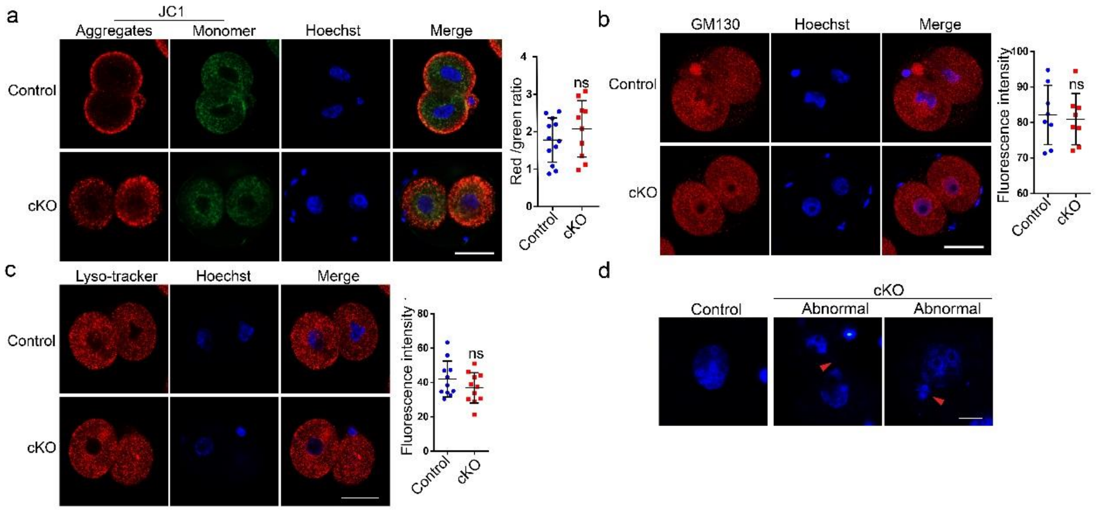

Figure S7. Preimplantation embryo development arrest of maternal *Ep400*-depleted embryos.

(a) JC1 staining in control and *Ep400*-depleted 2-cell embryos to detect MMP. MMP level is indicated by red/green ratio. Data are presented as means ± SD (n=12). Two-tailed student's t-test was used to calculate *p* values. Scale bar, 25µm. ns, not significant.

(b) GM130 staining in control and *Ep400*-depleted 2-cell embryos to show Golgi apparatus. Data are presented as means ± SD (n=8). Two-tailed student's t-test was used to calculate *p* values. Scale bar, 25µm. ns, not significant.

(c) Lysosome distribution in control and *Ep400*-depleted 2-cell embryos. Data are presented as means ± SD (n=11). Two-tailed student's t-test was used to calculate *p* values. Scale bar, 25µm. ns, not significant.

(d) Nuclear morphology of *Ep400* depleted 2-cell embryos. Red arrowheads indicate abnormal nuclei. Scale bar, 25µm.

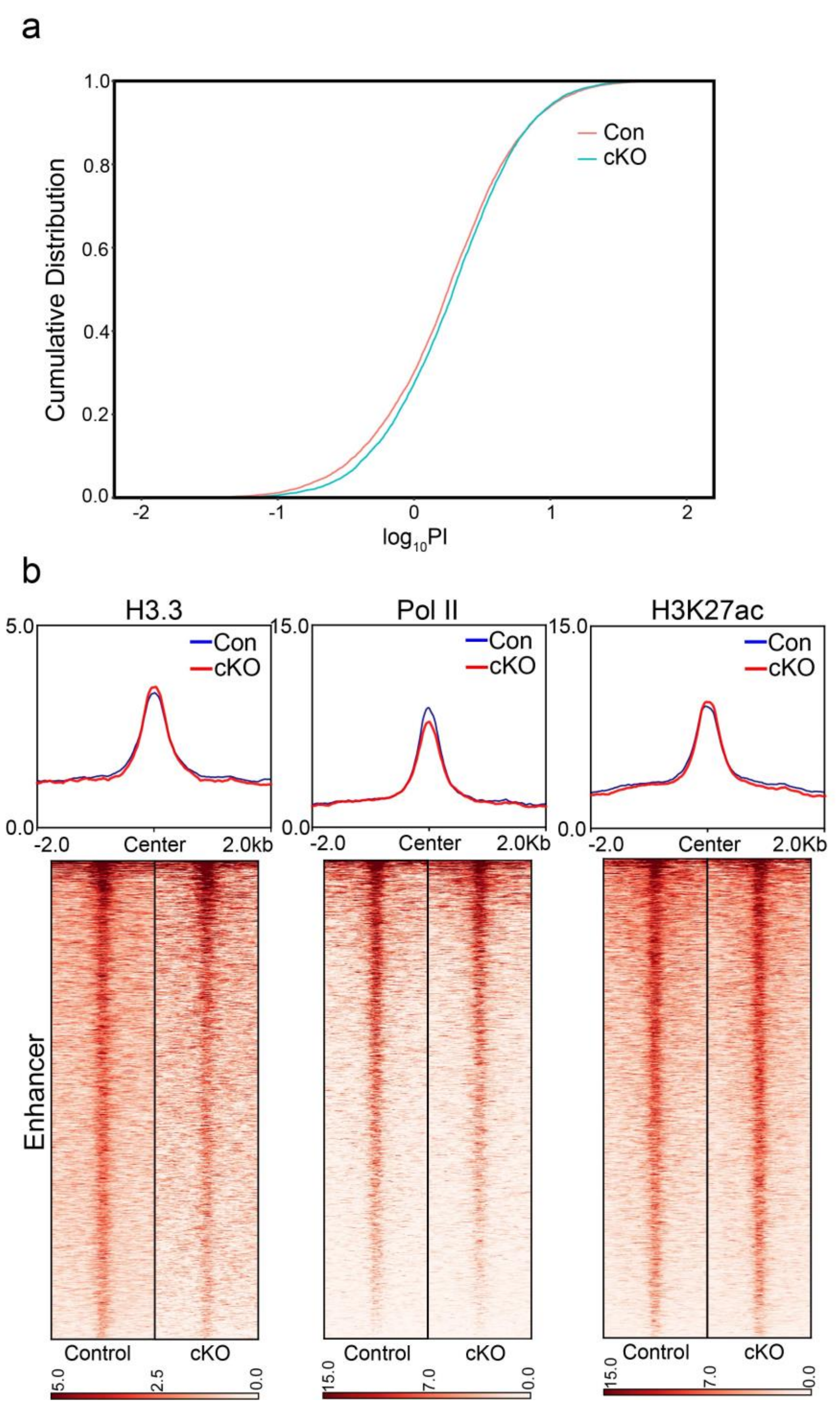

Figure S8. Impact of maternal *Ep400* on epigenetic modifications at late 2-cell stage.

(a) Cumulative curve illustrates increased PI value in cKO group relative to control group.

(b) Density plot of H3.3, Pol II, and H3K27ac around enhancer (H3K27ac-marked regions) and 2 kb upstream/downstream of enhancer in control and maternal *Ep400*-depleted late 2-cell embryos (upper panel), and corresponding heatmap (lower panel).

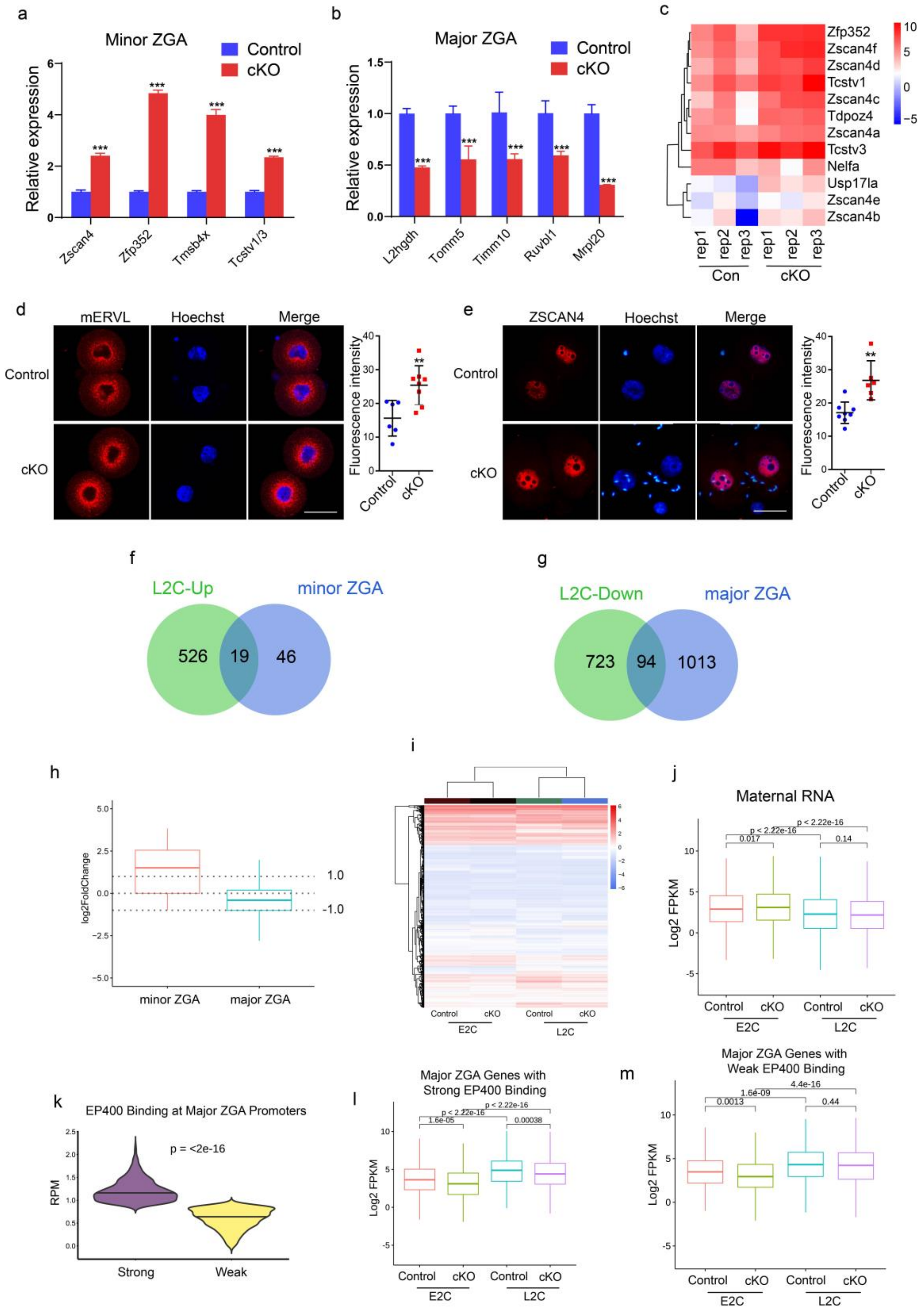

Figure S9. *Ep400* deficiency impacted expression of minor and major ZGA genes.

(a) Relative mRNA level of minor ZGA genes in late 2-cell embryos from control and cKO mice by qRT-PCR. Data are presented as means ± SD (n=3). mRNA levels of genes were normalized to *Actb*. Two-tailed student's t-test was used to calculate *p* values. ∗∗∗ $p < 0.001$.

(b) Relative mRNA level of major ZGA genes in late 2-cell embryos from control and cKO mice by qRT-PCR. Data are presented as means ± SD (n=3). mRNA levels of genes were normalized to *Actb*. Two-tailed student's t-test was used to calculate *p* values. ∗∗∗ $p < 0.001$.

(c) Heatmap of expression ($\log_2$FPKM) of representative upregulated ZGA genes in late 2-cell embryos.

(d-e) mERVL (d) (n=7) and ZSCAN4 (e) (n=8) staining of control and maternal *Ep400*-depleted late 2-cell embryos. Data are presented as means ± SD. Two-tailed student's t-test was used to calculate *p* values. Scale bar, 25μm. ∗∗ $p < 0.01$.

(f) Overlapping of minor ZGA genes and upregulated genes in late 2-cell embryos.

(g) Overlapping of major ZGA genes and downregulated genes in late 2-cell embryos.

(h) Log_2 (fold change) of minor and major ZGA genes at late 2-cell stage.

(i) Heatmap and clustering of transcriptome in early/late 2-cell embryos from control/cKO group.

(j) Comparison of expression changes of Maternal RNA in early/late 2-cell embryos from control/cKO group by RNA-seq data. Mann-Whitney U test was used to calculate *p* values.

(k) Major ZGA genes were evenly divided into two groups based on EP400 enrichment at gene promoters. Mann-Whitney U test was used to calculate *p* values.

(l) Expression of Major ZGA genes with strong EP400 association by RNA-seq data. Note that these genes had significantly reduced gene expression at late 2-cell stage in absence of maternal *Ep400*. Mann-Whitney U test was used to calculate *p* values.

(m) Expression of Major ZGA genes with weak EP400 association by RNA-seq data. Note that these genes had no evident changes of gene expression at late 2-cell stage in absence of maternal *Ep400*. Mann-Whitney U test was used to calculate *p* values.

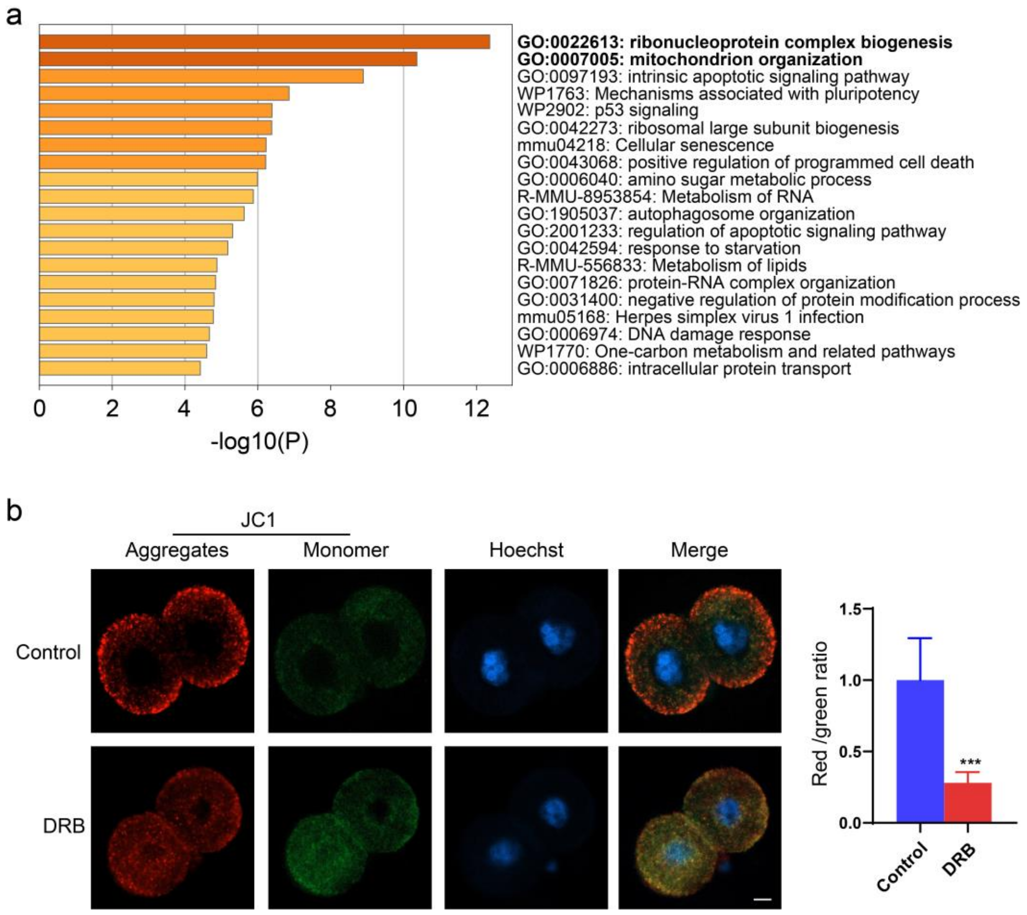

Figure S10. Major ZGA regulates mitochondrial functions.

(a) Gene ontology analysis of major ZGA genes.

(b) JC1 staining in late 2-cell embryos treated with or without DRB to detect MMP. MMP level is indicated by red/green ratio. Data are presented as means ± SD (n=7). Two-tailed student's t-test was used to calculate *p* values. Scale bar, 10μm. ∗∗∗ $p < 0.001$.

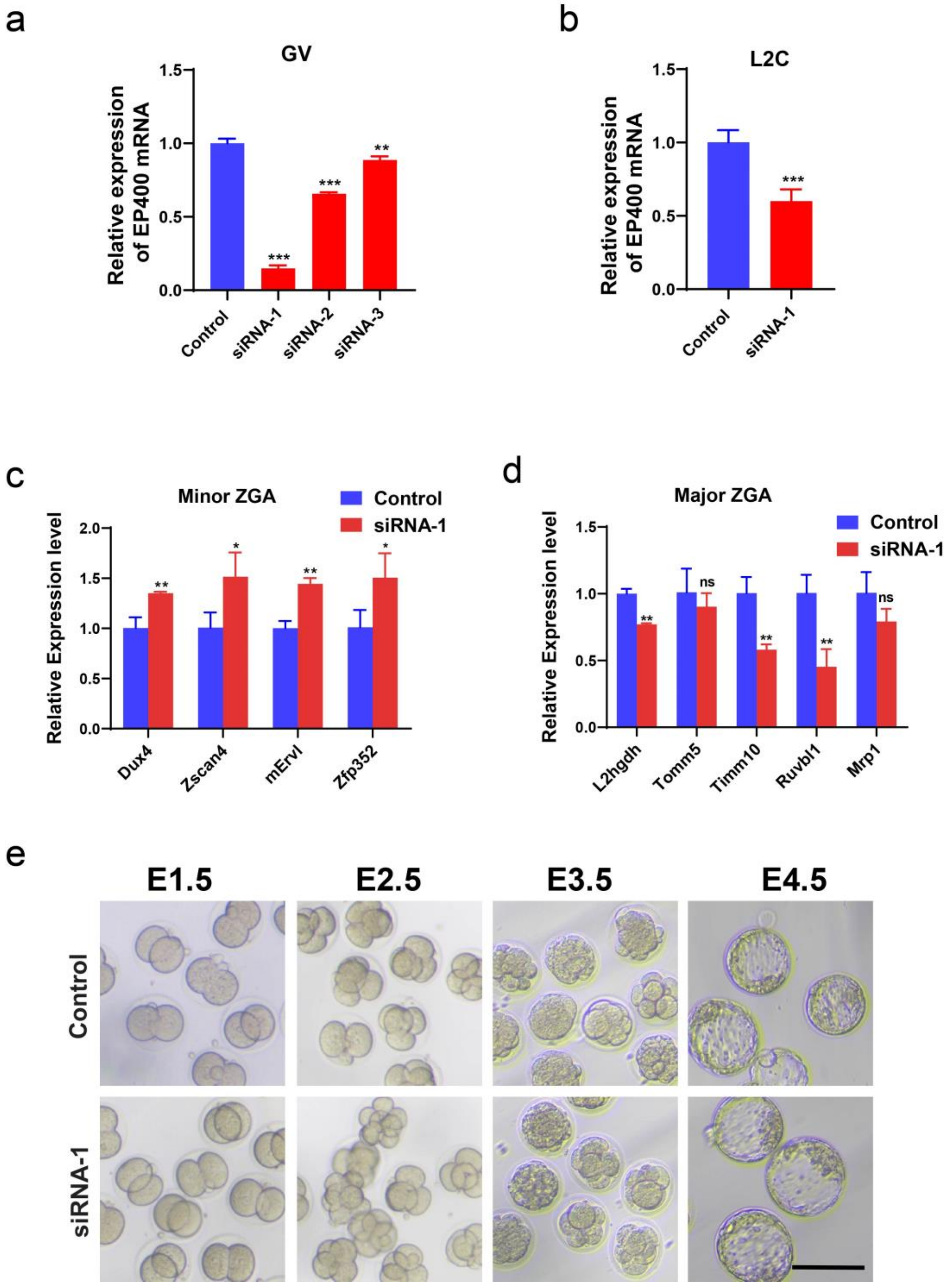

Figure S11. Knockdown of *Ep400* in early mouse embryos led to reduced expression of Major ZGA genes.

(a) Mouse GV oocytes were injected with siRNAs against *Ep400*, followed by oocyte collection at 48h post culturing in IVM medium with Milrinone for knockdown efficiency analysis by qRT-PCR. Data are presented as means ± SD (n=3). mRNA levels of genes were normalized to *Actb*. Two-tailed student's t-test was used to calculate *p* values. $**p < 0.01$, $***p < 0.001$.

(b-d) Mouse zygotes were injected with siRNA against *Ep400*, followed by embryo collection at late 2-cell stage. *Ep400* knockdown efficiency (b), typical Minor ZGA genes (c), and typical Major ZGA genes (d) were examined by qRT-PCR for relative gene expression. Data are presented as means ± SD (n=3). mRNA levels of genes were normalized to *Actb*. Two-tailed student's t-test was used to calculate p values. ns, not significant, $*p < 0.05$, $**p < 0.01$.

(e) Representative images of different developmental stages of embryos obtained from zygotes with injected siRNA (control, control siRNA; siRNA-1, siRNA-1 against *Ep400*) and cultured in KSOM medium. Scale bar, 100μm.

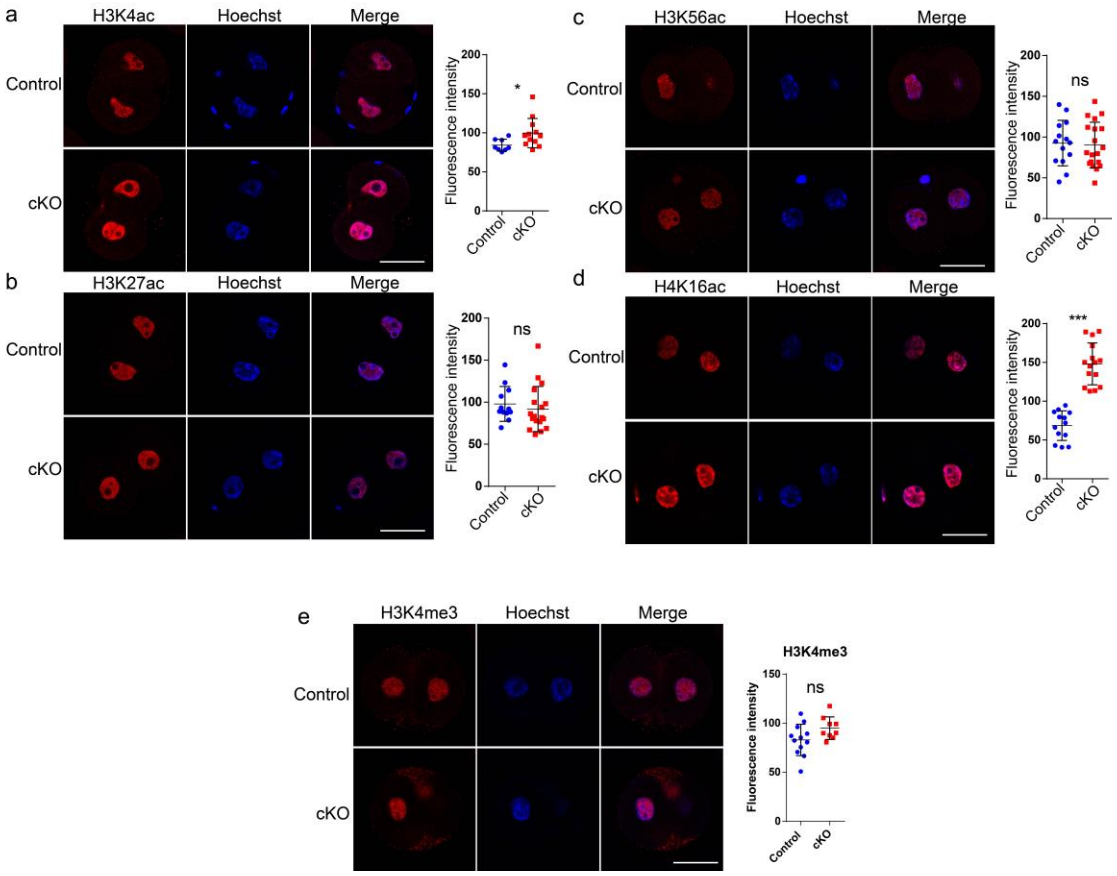

Figure S12. Effect of *Ep400* depletion on histone modifications in late 2-cell embryos.

(a-e) H3K4ac (a) (n=9), H3K27ac (b) (n=12), H3K56ac (c) (n=14), H4K16ac (d) (n=13) and H3K4me3 (e) (n=12) staining of control and maternal *Ep400*-depleted 2-cell embryos. Data are presented as means ± SD. Two-tailed student's t-test was used to calculate p values. Scale bar, 25μm. ns, not significant, $*\ p < 0.05$, $***\ p < 0.001$.

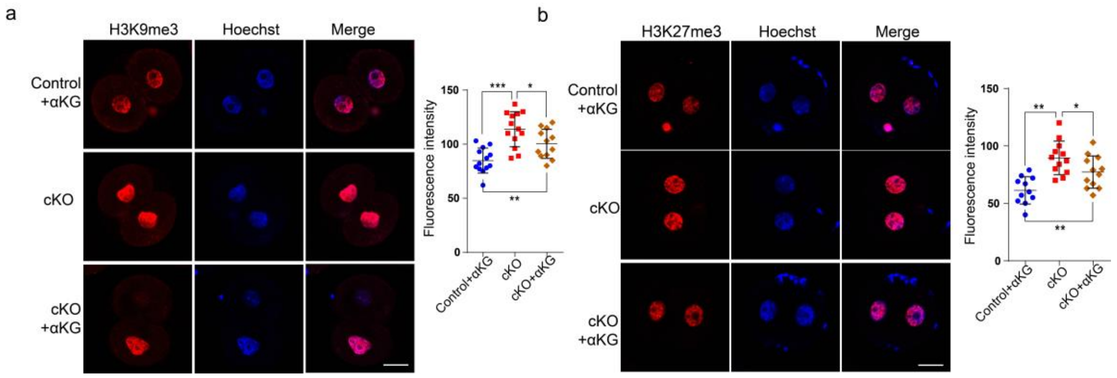

Figure S13. α-KG supplementation partially rescued histone methylation at late 2-cell stage. H3K9me3 (a) (n=13) and H3K27me3 (b) (n=12) staining in control with α-KG group, cKO group and cKO with α-KG group in late 2-cell embryos. Data are presented as means ± SD. Two-tailed student's t-test was used to calculate *p* values. Scale bar, 25μm. $* \ p < 0.05$, $** \ p < 0.01$, $*** \ p < 0.001$.

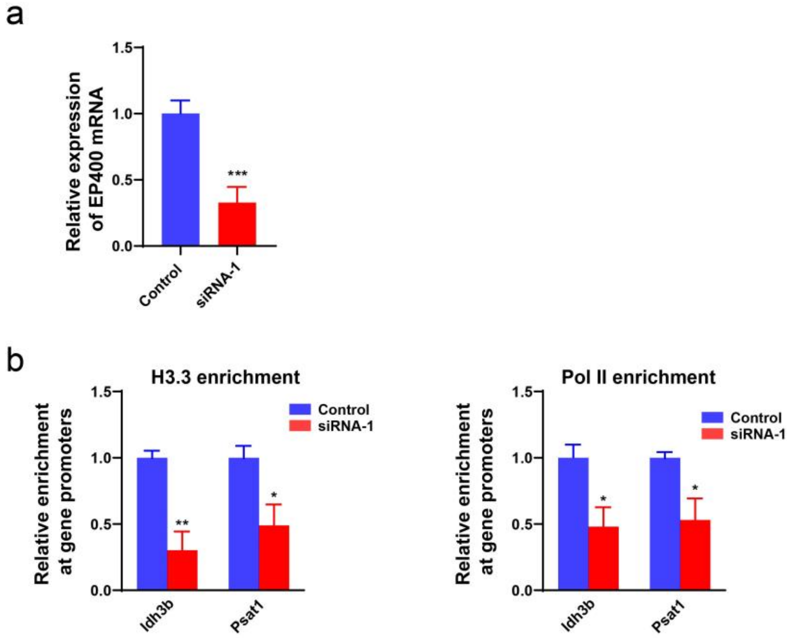

Figure S14. *Ep400* deficiency led to reduced enrichment of H3.3 and Pol II at *Idh3b* and *Psat1* promoters in mouse ESC.

(a) qRT-PCR was performed to verify knockdown efficiency at 48h post transfection of siRNA-1 against *Ep400* in mouse ESC. Data are presented as means ± SD (n=3). mRNA level of *Ep400* was normalized to *Actb*. Two-tailed student's t-test was used to calculate *p* values. $***p < 0.001$.

(b) ChIP-qPCR was performed to examine H3.3 and Pol II enrichment at *Idh3b* and *Psat1* promoter regions in control and *Ep400*-knockdown ESC. qPCR signals from ChIP samples were normalized to that of respective Input samples. Data are presented as means ± SD (n=3).

Two-tailed student’s t-test was used to calculate *p* values. $*p < 0.05$, $**p < 0.01$.

Supplementary materials

Table S1. Polypeptides table

Protein Name	Unique Peptides (anti-EP400)	Unique Peptides (IgG)
Bait: EP400	17	0
TRRAP	23	0
DMAP1	5	0
BRD8	4	0
PML	2	0
KDM1A	2	0
MBTD1	2	0
RNF2	2	0
ESRRB	4	2
OCT4	1	0
ING3	1	0
SIRT1	1	0

Table S2. Primer table

Primer	Sequence (5’-3’)
Ep400-Fl/Wt-F	ATTGGAAAATCCAACACCAAGGA
Ep400-Fl/Wt-R	GTCTCGGAGAGCACCATACAACAAAGATGG
Ep400-Del-R	CCCTGGGATGCCTGCAAGCTTATAACTTCG
Zp3-Cre-F	GGACATGTTCAGGGATCGCCAGGCG
Zp3-Cre-R	CCATGAGTGAACGAACCTGG
Ep400-F	AGATAGGCCTGGACTGGTTG
Ep400-R	GTTTTGAGGCCAGGACACCA
Timm10-F	TCCACGGTGCTAAGTTGACA
Timm10-R	GGCACTGGTCATTCTGTTGT
Tomm5-F	CTGCTGCGAGTCACTCCATA
Tomm5-R	TTCATGCCATGCATCCACCT
Mrpl20-F	CCCGAAACTTGGCCTCTGTA
Mrpl20-R	TGGGCATCAGTTGACCATGAA
Ruvbl1-F	GGACCATGCTATACACGCCA
Ruvbl1-R	AGCTGCACCGAATACCTCAG
L2hgdh-F	GCCTGCAGAATGGAGTCGAA
L2hgdh-R	TAGCCATTAGACCCCTGCAAT
Zscan4-F	GAGATTCATGGAGAGTCTGACTGATGAGTG
Zscan4-R	GCTGTTGTTTCAAAAGCTTGATGACTTC
Zfp352-F	ACCACCTCAAAGAACACCAG
Zfp352-R	ACAAGGGACAAGCGTAGAAC

Tcstv1/3-F	CCTTGCCTGTGATTCCGTCT
Tcstv1/3-R	ATCCTCCACCTCAGGCTGC
Tmsb4x-F	CCTCTGCCTTCAAAAGAAACAAT
Tmsb4x-R	AAAGGGGCAGCACAGTCATT
P53-F	CTCCGTCATGTGCTGTGACT
P53-R	GCAACTATGGCTTCCACCTG
Sesn2-F	CCTTCCGTGCCCAGGATTAT
Sesn2-R	TCTGATGCCAAAGACGCAGT
Tsc2-F	CAGGTGTGCAGAAGGCAAAC
Tsc2-R	CTCCACTGCATGCTCCTCAA
Ccng1-F	TGCTACACCAGCTGAACACC
Ccng1-R	AGATGCTTCGCCTGTACCTTC
Espl1-F	CTCAAGCCGCGACTTTTGC
Espl1-R	TCCTTCAAGTCAGGCAGCAG
Actb-F	GGCTGTATTCCCCTCCATCG
Actb-R	CCAGTTGGTAACAATGCCATGT
ND1-F	CTAGCAGAAACAAACCGGGC
ND1-R	CCGGCTGCGTATTCTACGTT
β-globin-F	GAAGCGATTCTAGGGAGCAG
β-globin-R	GGAGCAGCGATTCTGAGTAGA
Idh3b-Promoter-For	AGGATGGGCGAGAACGAAGC
Idh3b-Promoter-Rev	TGGGCGGGAGAACAGAGGGT
Psat1-Promoter-For	TCGGAGTCTACGATCAATCAA
Psat1-Promoter-Rev	GGACTGGCTCTGGGACAAAT

Table S3. Antibody table

Antibody	Source	Product code
Anti-TOM20 antibody	Abcam	ab78547
Anti-GM130 antibody	Abcam	ab52649
Anti-γH2A.X antibody	Abcam	ab26350
Anti-RAD51 antibody	Abmart	T62573S
Anti-53BP1 antibody	HUABIO	ET1704-05
Anti-PARP1 antibody	Proteintech	12981-1-AP
Anti-α-tubulin antibody	Sigma	SAB4500087
Anti-ZSCAN4 antibody	Novus	NBP2-75722
Anti-EP400 antibody	Bethyl	A300-541A
Anti-EP400 antibody (ChIP-seq in mESC)	Abcam	ab70301
Anti-PolII antibody	Active Motif	39097
Anti-H3K9me3 antibody	Active Motif	39161
Anti-H3.3 antibody	Active Motif	91191
Anti-H3K4me3 antibody	CST	9727
Anti-H3K27ac antibody	Abcam	ab177178
Anti-H3K27me3 antibody	Diagenode	C15410069
Anti-H3K4ac antibody	Active Motif	39381

Anti-H3K56ac antibody	Active Motif	39281
Anti-H3K64ac antibody	Abcam	ab214808
Anti-H4K16ac antibody	Abcam	ab109463
Anti-ERVL antibody	Novus	NBP2-66963
Anti-NFYA antibody (for WB)	Proteintech	12981-1-AP
Anti-NFYA antibody (for CoIP)	Abmart	PA6243
Anti-ESRRB antibody	Proteintech	22644-1-AP
Anti-OCT4 antibody	Santa Cruz	sc-5279
Anti-IDH3B antibody	ABclonal	A13742
Anti-PSAT1 antibody	Proteintech	10501-1-AP
Anti-Ovastacin antibody	Gift from Dr. Bo Xiong	N/A
Goat anti-mouse IgG (H+L)	Yeasen	33912ES60
Goat anti-rabbit IgG (H+L)	Yeasen	33112ES60
